# Coverage of Adequately Iodized Salt Is Suboptimal and Rice Fortification Using Public Distribution Channels Could Reach Low-Income Households: Findings from a Cross-Sectional Survey of Anganwadi Center Catchment Areas in Telangana, India

**DOI:** 10.1371/journal.pone.0158554

**Published:** 2016-07-22

**Authors:** James P. Wirth, Magali Leyvraz, Prahlad R. Sodani, Grant J. Aaron, Narottam D. Sharma, Bradley A. Woodruff

**Affiliations:** 1 GroundWork, Fläsch, Switzerland; 2 Global Alliance for Improved Nutrition, Geneva, Switzerland; 3 Indian Institute of Health Management Research, Jaipur, India; RTI International, UNITED STATES

## Abstract

Food fortification is a cost-effective approach to prevent and control of micronutrient deficiencies in India. A cross-sectional survey of children 0–35 months of age residing in the catchment areas of anganwadi centers in the state of Telangana was conducted to assess the coverage of adequately iodized salt and the potential for rice fortification. Salt samples were collected and tested for iodine concentration using iodometric titration. Information on demographics, household rice consumption, and Telangana’s rice sector was collected and interpreted. In households of selected children, 79% of salt samples were found to be adequately iodized. Salt brand and district were significant predictors of inadequately iodized salt. Daily rice consumption among children and women averaged 122 grams and 321 grams per day, respectively. Approximately 28% of households reported consuming rice produced themselves or purchased from a local farmer, 65% purchased rice from a market or shop, 6% got rice from a public distribution system site, and 2% obtained it from a rice mill. In the catchment areas of Telangana’s anganwadi centers, there is significant variation in the coverage of adequately iodized salt by district. Future surveys in Telangana should measure the coverage of salt iodization in the general population using quantitative methods. Nonetheless, increasing the adequacy of iodization of smaller salt manufacturers would help achieve universal salt iodization in Telangana. Despite high consumption of rice, our findings suggest that large-scale market-based rice fortification is not feasible in Telangana due to a large proportion of households producing their own rice and highly fragmented rice distribution. Distributing fortified rice via Telangana’s public distribution system may be a viable approach to target low-income households, but would only reach a small proportion of the population in Telangana.

## Introduction

Due to its large population and high burden of malnutrition, India has the highest number of children born vulnerable to iodine deficiency [1] and the greatest number of stunted children [2] in the world. Forty percent of the world’s children born with low birth weight (<2.5 kg) are born in India [3]. Indian women are burdened by multiple micronutrient deficiencies [4], and more than half of India’s women are anemic [5]. An estimated 1% of India’s gross domestic product is lost annually due to micronutrient deficiencies [6]. While India has made strides in documenting the nutrition status of the population, there remains a dearth of nutrition-related information at the state and district levels.

Globally, the fortification of staple foods with micronutrients is recognized as an affordable and effective approach to reducing and preventing vitamin and mineral deficiencies [7,8]. In India, food fortification is used to improve the micronutrient content of multiple sample foods, including salt and rice [9,10]. In this paper, we investigate the coverage of *adequately* iodized salt and the potential of fortifying rice in populations served by the Integrated Child Development Services (ICDS) in Telangana, a state established on June 2014, following the division of Andhra Pradesh into two states: Telangana and Seemandhra.

From 2010 to 2014, the Global Alliance for Improved Nutrition (GAIN) supported the manufacture and distribution of a complementary take-home ration (Bal Amrutham) to children in Telangana through the ICDS program GAIN also supported staple food fortification efforts in other Indian states. A survey was undertaken to assess the coverage and utilization of Bal Amrutham in 2014, and (results of this work are presented in another paper in this supplement [11]). This survey had as a secondary objective to assess the coverage of adequately iodized salt and consumption and distribution of rice in ICDS-served populations, and this paper presents these findings.

### Salt iodization in India

India’s national government began efforts to promote the consumption of iodized salt in the 1960s [9,12]. Following decades of promotion, the iodization of salt for human consumption was made mandatory in 1997 and again in 2005. Mandatory salt iodization legislation was repealed in 2000, when the sale of *non*-iodized salt was permitted [12]; the repeal remained in effect until 2004. According to India’s 2009 Coverage Evaluation Survey, 90.4% of household salt samples were iodized, and 71.1% were adequately iodized, that is, they contained at least 15 parts per million (ppm) of iodine [13]. Despite this relatively high coverage, low-income and rural households were less likely to consume adequately iodized salt [14]. Prior to the division of Andhra Pradesh, the coverage of adequately iodized salt in the state was 63.6%—below the national average. To further expand the coverage of adequately iodized salt, researchers and policy makers have recommended that governments reinforce quality assurance/control systems and routinely monitor household coverage of adequately iodized salt to identify socioeconomic groups and geographic locations where salt iodization can be improved [15].

### Rice fortification in India

Whereas multiple state-level programs in India fortify wheat flour and vegetable oil [16], rice-fortification programs are in their infancy. In areas in India where rice is a staple food, rice fortification is being explored and tested as an intervention to reduce micronutrient deficiencies. Fortified rice has been shown to decrease iron deficiency in India [17], and small-scale rice-fortification projects have been conducted in Odisha [10] and in present-day Seemandhra [18]. Telangana produces several rice varieties, and rice is the main staple food consumed in all areas of Telangana.

Around the world, rice fortification has proved to be an effective strategy for improving vitamin and mineral intakes and status [19,20], thereby reducing micronutrient deficiencies [17,21] and anemia [20,22]. In recent years, many of the technical challenges of implementing rice-fortification programs have been overcome, and large-scale rice-fortification projects have been implemented in Brazil, Indonesia [23], Costa Rica, and the Philippines [24]. While the potential of rice fortification to address micronutrient deficiencies has been established, implementing rice fortification on a large scale is feasible only if specific criteria are met. In particular, market fragmentation and rice processing capacity are formidable barriers to the implementation of large-scale market-based rice-fortification programs [25].

## Materials and Methods

### Study design

We conducted a two-stage stratified cross sectional survey in Telangana in November—December 2014. The survey’s primary objective was to estimate the coverage of a fortified complementary food distributed by Telangana’s anganwadi centers (AWCs), which are local public health and nutrition centers operating under the ICDS. AWCs served as the primary sampling unit of the survey, and sampling was stratified by grouping AWCs into two general categories, urban and rural, based on their location. At the time of the survey, there were 35,553 AWCs operating in Telangana; 3,756 in urban areas and 31,797 in rural areas. AWCs were randomly selected with equal probability in the first stage of sampling. In the first stage of sampling, 45 AWCs were randomly selected per stratum with equal probability; 90 AWCs were selected in total. Following a census of all children under 3 years old living in the catchment area of each selected AWC, the second stage of sampling consisted of the selection of 13 children using simple random sampling. No replacement of unavailable children was conducted to avoid selection bias within the sample. As a result of this sampling scheme, all children living in the catchment area of a selected AWC were eligible for selection, not only children enrolled in the AWC at the time of sampling.

Survey results are not representative of the overall Telangana population, but rather are representative only of children living in areas covered by the ICDS program. Questionnaires (S1 File) were administered to the mother or caregiver of each selected child. In addition to assessing the coverage of fortified-complementary food distributed to young children by the AWCs, the questionnaire also collected information on household demographic variables, household purchases, and consumption of salt and rice.

### Collection and analysis of salt samples

Interviewers collected about 20 grams of salt from each household. The sample was placed in a small plastic bag, labeled, and placed in a larger plastic bag to prevent spillage during transport and handling and then placed in an opaque bag or pouch to prevent exposure to light. Iodometric titration was carried out as per the standard guidelines to estimate iodine content of each salt sample [26]. The analysis was conducted by the International Council for the Control of Iodine Deficiency Disorders (ICCIDD) regional reference laboratory in New Delhi. Quality control charts (Levi-Jenning Plots) were prepared by testing known value samples. Before running the test each day, known value samples were analyzed to assess the accuracy of the procedure. Overall, there was a coefficient of variation (CV) for duplicates of a maximum of 10%. Salt containing ≥15 ppm of iodine were considered adequately iodized [15].

### Ethics and consent

This survey protocol was reviewed and approved by the ethical review committee of the Indian Institute of Health Management Research. At each selected household, the advantages and risks for participating household members were explained, and oral informed consent was obtained from participating caregivers, with the selected child’s mother or legal guardian giving oral consent for her/his child selected for data collection.

### Data management

Multiple reviews of completed questionnaires were conducted in the field by the field editors and team leader. After transport to headquarters, questionnaires were double entered and cross-checked using CSPro v. 5.0 (United States Census Bureau, Washington, DC, USA). Poverty was assessed using a multidimensional poverty index (MPI) [27], whereby living standards, education, health and nutrition, and household assets are combined to create an index ranging from 0 to 1, where 0 indicates no poverty. A household is defined as being at risk for poverty (poor) if its MPI is ≥0.33, and a household is defined as being *not* at risk of poverty (non-poor) if its MPI is <0.33.

Statistical analyses were conducted using SPSS (version 21, IBM Corporation, Armonk, NY, USA). Results were weighted to correct for unequal probability of selection among strata using sampling weights calculated from the total number of children under 36 months in each AWC catchment area. Continuous data were checked for skewness using the Cox test (coefficient of skewness divided by the standard error of skewness), as well as by examination of the frequency distribution. The statistical significance of differences in categorical variables between subgroups was judged using the chi-square test and in continuous variables by independent-sample t-test or one-way analysis of variance (ANOVA). P-values less than 0.05 were considered statistically significant.

### Analysis approach: salt

We assessed the coverage of adequately iodized salt by stratum, district, MPI status, caregiver education, and brand and/or type of salt. Due to the large number of salt brands reported, the six most widely reported brands are referred to here as “major brands 1–6” and are presented separately. Less-frequently reported brands are listed as “other.” As some respondents purchased “open salt” (salt sold by weight from a large bag) or did not know the brand of salt, “open salt” and “don’t know” options were also assessed. Variables found to be statistically significantly associated with the household presence of adequately iodized salt were included in a logistic regression model to measure their strength of association independent of confounding from other variables. For the regression model, brand and/or type of salt was recoded into four summary subgroups—“major brand,” “other brand,” “open salt,” and “don’t know brand”—because the large number of individual subgroups for salt brand and/or type of salt led to quasi-complete separation of the model.

### Analysis approach: rice

We explored the feasibility of rice fortification in Telangana using a simplified set of criteria based on the multiple “success factors” for rice fortification described by Piccoli et al [25]: 1) need (i.e., micronutrient deficiencies exist in the population), 2) sufficient consumption, 3) marketing and distribution capacity, and 4) technical and logistical capacity.

Literature related to anemia and micronutrient deficiencies in Telangana and nearby states was investigated to determine if fortification in Telangana is needed and justifiable. To estimate daily rice consumption in grams for children 6–59 months and female caretakers, we used: 1) the number of adult male equivalents (AMEs) in each household calculated from a household roster [28,29], 2) the daily quantity of rice consumed at the household calculated using responses to interview questions about the amount of the most recent purchase and how long this quantity usually lasts, and 3) consumption for selected children and caretakers calculated as a proportion of household daily rice consumption matching the individual’s proportion of the total household AMEs. Using the average consumption of these groups, we estimated the proportion of recommended dietary allowance (RDA) that would be achieved using a) the staple food fortification levels recommended by India’s National Institute of Nutrition [30] and b) the nutrient requirements of the Indian population [31]. According to India's fortification recommendations, fortified rice needs to contain "one or more of the following materials" at the specified levels per kilogram of rice: calcium (1500 mg), iron ferric pyrophosphate (60mg), zinc (30 mg), vitamin A (1500 μg Retinol Equivalents), vitamin C (100 mg), thiamine (3.5 mg), riboflavin (4.0 mg), niacin (45 mg), vitamin B_6_ (5 mg), folic acid (250 μg), and vitamin B_12_ (2.5 μg) [30]. The RDA of iron from conventional and fortified rice was calculated assuming that 5% bioavailability [31].

To assess marketing and distribution capacity, data on rice brands consumed were assessed to determine if the rice market is consolidated and if there are any large companies covering a substantial proportion of the market. To assess technical and logistical capacity of fortifying rice, we reviewed literature related to programmatic experiences of rice fortification in India and elsewhere.

## Results

### Adequacy of iodized salt

The iodine concentrations found in the salt samples ranged from 0–174 ppm. Only 6 samples were found with 0 ppm iodine, thus, 99% of salt samples can be considered iodized. Over-iodization was also rare, with only 2.3% of samples (n = 28) displaying iodine concentrations >50 ppm.

On average, 78.8% of the salt samples in the households of children living in AWC catchment areas were adequately iodized (Table 1). A significantly greater proportion of adequately iodized salt was observed in urban areas (p<0.05), households classified as non-poor (p<0.01), and households with caregivers with ≥5 years of schooling (p<0.01). District-level analysis showed a wide variation in the coverage of adequately iodized salt in AWC catchment areas, ranging from 59.0% in Nizamabad district to 94.1% in Medak district. Nearly half of all households consumed salt from the six major brands. The remaining households either consumed salt from another brand (25.6%) or did not know the brand (25.1%).

**Table 1:** Percentage of Adequately Iodized Salt (≥15 ppm Iodine Concentration) in Households of Selected Children (N = 1068) in Selected AWC Catchment Areas in Telangana, India, 2014.

Characteristic	n	%^a^	(95% CI)^b^	P-value^c^
**Residence**				
Urban	470	87.8	(82.6, 91.6)	<0.05
Rural/Tribal	400	77.0	(69.0, 83.5)	
**MPI**				
Poor	128	66.2	(53.4, 77.0)	<0.01
Non-poor	712	82.7	(76.2, 87.7)	
**Education of caregiver (years)**				
0–4	217	71.2	(59.1, 80.9)	<0.01
≥5	653	83.7	(78.7, 87.6)	
**District**				
Adilabad	127	84.5	(75.9, 90.4)	<0.01
Hyderabad	104	92.3	(83.0, 96.7)	
Karimnagar	88	90.5	(78.5, 96.1)	
Khammam	132	82.5	(63.2, 92.8)	
Mahbubnagar	106	65.5	(45.9, 81.0)	
Medak	38	94.1	(89.6, 96.8)	
Nalgonda	79	78.5	(65.3, 87.7)	
Nizamabad	33	59.0	(39.9, 75.8)	
Ranga Reddy	61	70.1	(32.7, 91.9)	
Warangal	102	71.9	(62.7, 79.6)	
**Brand**				
Major brand 1	129	92.0	(83.9, 96.2)	<0.001
Major brand 2	77	94.6	(88.1, 97.7)	
Major brand 3	80	82.0	(64.6, 91.9)	
Major brand 4	62	100.0	–	
Major brand 5	43	86.6	(76.8, 92.6)	
Major brand 6	142	94.6	(87.2, 97.8)	
Other brands	155	64.5	(50.0, 76.8)	
Don’t know	178	71.3	(64.0, 77.6)	
Open salt	3	13.8	(3.70, 40.0)	
**TOTAL**	870	78.8	(72.1, 84.2)	–

CI, confidence interval.

Note: The n’s are unweighted numbers with the condition (i.e., numerator) for each subgroup; subgroups that do not sum to the total have missing data.

^a^ Percentages weighted for unequal probability of selection.

^b^ CI calculated taking into account the complex sampling design.

^c^ Chi-square p-value <0.05 indicates that the variation in the values of the subgroup is significantly different from all other subgroups.

In a logistic regression model, only district and salt brand and/or type were significant predictors of inadequately iodized salt in AWC catchment areas (Table 2). Using Hyderabad and Medak as references, the odds of having inadequately iodized salt is three times higher in AWC catchment areas in Nalgonda, four times higher in Warangal, almost five times higher in Mahbubnagar, and about six times higher in Ranga Reddy and Nizamabad. The odds of having inadequately iodized salt were 40 times higher in open salt, seven times higher for other brands, and five times higher in salt with an unknown brand.

**Table 2:** Logistic Regression Odds Ratios for *Inadequately* Iodized Salt Using Factors with Statistically Significant Associations in Households of Selected Children (N = 1068) in Selected AWC Catchment Areas in Telangana, India, 2014.

Characteristic	n	Odds ratio	(95% CI)^b^	P-value
**Residence**				
Urban	525	Reference	–	0.34
Rural/Tribal	507	1.3	(0.76, 2.2)	
**MPI**				
Non-poor	852	Reference	–	
Poor	180	1.5	(0.64, 3.4)	0.35
**Education of caregiver (years)**				
≥5	746	Reference	–	
0–4	286	1.2	(0.57, 2.4)	0.67
**District**				
Hyderabad/Medak^a^	147	Reference	–	<0.01
Adilabad	162	2.7	(1.1, 6.9)	
Karimnagar	91	1.1	(0.36, 3.50)	
Khammam	153	1.8	(0.49, 6.20)	
Mahbubnagar	137	4.8	(1.80, 13.0)	
Nalgonda	92	3.1	(1.4, 7.3)	
Nizamabad	49	5.9	(2.30, 15.4)	
Ranga Reddy	72	5.5	(1.60, 18.8)	
Warangal	129	4.4	(1.9, 9.9)	
**Salt type**				
Major brands	560	Reference	–	<0.001
Open salt	20	40.9	(9.5, 176.9)	
Don’t know brand	235	5.0	(2.6, 9.8)	
Other brands	217	7.1	(3.3, 15.4)	

CI, confidence interval.

Note: The n’s are unweighted numbers with the condition (i.e., numerator) for each subgroup; subgroups that do not sum to the total have missing data.

^a^ Hyderabad and Medak considered as one group to prevent quasi-separation of model.

^b^ CI adjusted for cluster sampling design.

### Feasibility of fortifying rice

Table 3 presents the main findings of each criterion used to examine the feasibility of fortifying rice in Telangana. Detailed findings for each criteria are detailed below.

**Table 3:** Feasibility of Rice Fortification in Telangana, India.

Prerequisite Category	Data Sources	Findings	Opportunities for Telangana
Need/documented micronutrient deficiencies	Peer-reviewed literature	High prevalence of anemia and vitamin A deficiency among children 6–59 months of age and pregnant women; high prevalence of zinc deficiency among children 6–59 months	No representative or current data in Telangana (or Andhra Pradesh) on anemia and iron, vitamin A, folate, or vitamin B12 deficiencies. More current evidence of micronutrient deficiencies is needed to justify rice fortification.
Sufficient consumption	2014 Telangana survey	High consumption among both children 6–35 months of age and women 15–49 years of age	Assuming fortification using recommended fortification standards, fortified rice would contribute ≥80% of the RDA for multiple micronutrients in women and ≥80% of RDA iron, thiamin, and riboflavin in children. Increased % RDA of vitamins A and B12 in children and women is substantial considering documented vitamin A deficiency and potential B12 deficiency.
Marketing potential and distribution capacity	2014 Telangana survey and literature on Telangana’s rice milling industry	Fractured commercial market, distribution via India’s public distribution system more feasible than market-based approach	Rice distribution to AWCs as part of midday meals program.
Technical and logistical capacity	Peer-reviewed literature related to rice fortification and programmatic reports from rice-fortification projects in India	Extrusion of fortified rice kernels already conducted in India and recommend by India’s National Institute of Nutrition	Rather than establish separate extrusion facilities in Telangana, procurement of fortified kernels from suppliers in India and elsewhere may be the most cost-effective approach.

#### Need/documented micronutrient deficiencies

Data on micronutrient deficiencies are available only for the pre-existing state, Andhra Pradesh, which may or may not apply to Telangana after Andhra Pradesh’s division into two states. In children, representative surveys have identified a high level of anemia and vitamin A and zinc deficiencies. In 2005–06, India’s National Family Health Survey found that 70.8% of children 6–59 months of age in Andhra Pradesh were anemic [32]. A survey conducted in 2000 found that 52.3% of children 12–47 months old in Andhra Pradesh were vitamin A deficient [33]. A study examining postnatal maternal vitamin A supplementation found that about 80% of Hyderabadi newborns were vitamin A deficient [34]. Among children 6–60 months of age selected from AWC catchment areas in Karnataka, which borders Telangana, 36.2% were zinc deficient [35].

Among women 15–49 years old in Andhra Pradesh, India’s 2005–06 National Family Health Survey found that 62.9% were anemic [32]. A 2006 study of untouchable caste (Dalit) members in Medak district examining symptoms of vitamin A deficiency found that 16% of women suffered from night blindness, Bitot’s spot, or conjunctival xerosis [36].

The soil in Andhra Pradesh has also been identified as highly deficient in zinc [37], and a relationship between zinc soil content, the content of zinc in rice, and subsequent zinc deficiency in women and children has been shown [38]. We could not identify any studies in Andhra Pradesh estimating iron deficiency or other micronutrient deficiencies (e.g. folate, vitamin B12) that can be addressed using rice fortification. Moreover, most data are more than 10 years old and thus do not accurately depict the current micronutrient status of children and women in Telangana.

#### Sufficient consumption and estimated nutrient intake from fortified rice

The estimated consumption of rice was high for both children 6–35 months of age and their caretakers in AWC catchment areas. Mean rice consumption among children and women was 122 grams (95% CI: 115, 128) and 321 grams (95% CI: 306, 336) per day, respectively. In both children and women, rice consumption was lowest in Hyderabad (97.7 grams in children; 260.2 grams in caretakers) and highest in Mahbubnagar (260.2 grams in children; 379.1 grams in caretakers). Rice consumption was significantly higher in rural areas, poor households, households with a less educated caretaker, and in Mahbubnagar and Warangal districts. These findings were consistent in both children and their caretakers.

**Fig 1:**
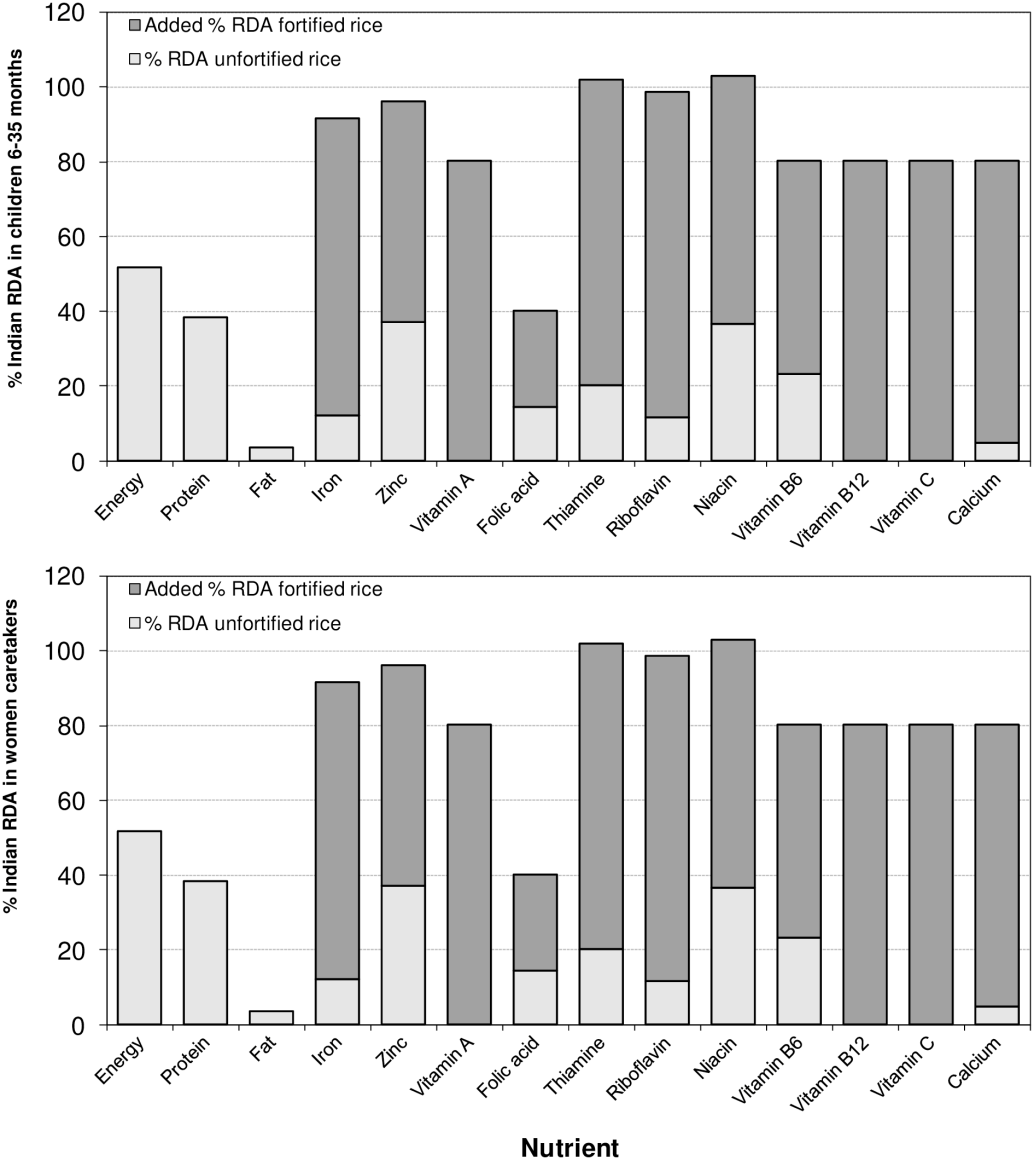
Percent of Indian RDA for Children and Their Caretakers Based on Rice Consumption Patterns in Households of Selected Children (N = 1068) in Selected AWC Catchment Areas in Telangana, India, 2014.

Fig 1 presents the percentage of RDA contributed by consumption of unfortified vs. fortified rice in children and women in Telangana AWC catchment areas. Children consuming fortified rice would have an intake of more than 80% of the RDA for iron, thiamin, and riboflavin. Women consuming fortified rice would have an intake ≥80% of the RDA of many more micronutrients: iron; zinc; thiamin; riboflavin; niacin; vitamins A, B6, B12, and C; and calcium. Because unfortified rice contains no vitamin A or vitamin B12, the total contribution of intake of these nutrients from rice must be from fortified rice, which would provide 80.2% of the RDA of both nutrients in women and 46.3% and 62.0%, respectively, of the RDA in children.

#### Marketing potential and distribution capacity

The marketing potential and distribution capacity in AWC catchment areas was assessed by asking caretakers where they procured rice from and if they knew the brand of the rice that they purchased. Among all respondents, 21.8% (95% CI: 15.7, 29.4) produced their own rice, while others procured rice; 64.5% (95% CI: 57.6, 70.9) purchased rice from a market or shop, 6.0% (95% CI: 4.0, 8.9) from a local farmer, 6.2% (95% CI: 4.2, 8.9) from a public distribution system (PDS) site, and 1.5% (95% CI: 0.8, 2.7) from a rice mill.

A slightly higher percentage of poor households procured rice from the PDS (10.4%) than did non-poor households (4.4%). No difference between PDS rice was observed among urban and rural households. Among those procuring rice, 86.7% (95% CI: 82.2, 90.2) did not know the brand. Not knowing the rice brand was highest among respondents from rural areas (88.3%) and poor households (92.6%). Of those purchasing branded rice, 66 unique brands of rice were identified.

#### Technical and logistical capacity

Although multiple approaches can be used to fortify rice, the use of hot or cold extruded rice kernels has been cited as more effective than dusting or coating technologies because dusting and coating rice with micronutrients results in organoleptic changes and the micronutrients can be removed during washing [39]. Extrusion is the rice fortification strategy recommended by India’s National Institute of Nutrition [30], and extruded rice has been used India’s pilot rice-fortification programs. Extrusion facilities in India have been used to produce fortified extruded rice kernels that do not affect the sensory qualities of the rice [18]. New extrusion facilities would not be required in Telangana, as rice fortification can be done by purchasing extruded kernels from external sources and blending them with local rice. Using this approach, rice fortification can be implemented in Telangana with considerable cost savings [40].

## Discussion

### Coverage of adequately iodized salt

The survey results from Telangana AWC catchment areas support the findings from national population-based surveys measuring salt iodine concentrations using semi-quantitative rapid test kits. These have shown a considerable increase in the coverage of adequately iodized salt in Andhra Pradesh over the past 15 years, from 27.5% in 1999 [41] to 31.0% in 2005 [32] and 63.6% in 2009 [13].

Despite these state-level improvements, analysis of the survey’s results suggests that significant variation in the coverage of adequately iodized salt in households in AWC catchment areas may occur between districts. While the survey results are not representative of the population or all households in Telangana, our findings were similar to those found by a representative survey conducted in Nalgonda district in 2014, which found 83% of household salt samples adequately iodize [42]. Nonethelss, future population- and household-representative iodine surveys in Telangana should estimate iodized salt coverage at the state and district levels. Such surveys should use quantitative analysis of salt iodine. Pandav and colleagues [43] have shown that salt iodine rapid test kits cannot accurately assess the iodine concentration in salt; they can assess only if salt contains any iodine. In addition to iodometric titration, there are several field-friendly devices that quantitatively measure salt iodine concentrations [44].

As salt is traded extensively within India, national-level efforts, such as legislation and quality assurance monitoring, are warranted. In additional to monitoring the iodine concentrations via national and state-representative surveys, India’s revised guidelines on iodine deficiency disorders recommend that district-representative surveys measuring the coverage of adequately iodized salt (among other indicators) should be conducted every 5 years [9,12]. The findings of this survey suggest that additional efforts to increase the adequacy of iodization of smaller salt manufacturers may be an efficient approach to achieving universal salt iodization in Telangana. As adequacy of salt iodization was lowest among companies with a smaller market share, future state- and district-level iodine assessment surveys should also collect information on the brand of salt to identify which companies are not in compliance with governmental standards. To increase the adequacy of salt iodized by smaller producers, the Telangana government and/or nutrition stakeholders should assess these producers' quality control procedures, procurement of potassium iodate, and the packaging, storage, and distribution practices used, to determine which part of the salt iodization process can be enhanced.

### Feasibility of fortifying rice in Telangana

While there is recent experience in fortifying rice in India, our findings suggest that market-based fortification would not be feasible under current conditions. A notable proportion of rural (25%) and poor (23%) respondents in AWC catchment areas reported that their household consumed rice that they produced themselves, which would preclude fortification. Moreover, rice distribution is highly fragmented even among rice with known brands. According to Alavi et al. [39], rice fortification is easiest to implement and monitor and is most cost efficient in mills producing approximately 15,000 metric tons per year. In 2014, it was reported that approximately 600 rice mills are members of the Telangana Rice Millers Association [45], suggesting a complex environment to implement market-based rice fortification. A 2016 report noted that there are 200 operational rice mills in Telangana, and large closures of rice mills due to reductions in the "levy rice" quota [46]. We could not find a detailed assessment of the rice millers in Telangana, and thus the current production capacity of each mill is unknown.

Telangana’s PDS, on the other hand, has the potential to cover about 2.1 million individuals based on a current population size of 35 million [47] and an estimated 6% of respondents receiving rice from the PDS. India’s PDS has been used to distribute fortified rice to school children and low-income households [23], and fortified rice could also be provided as part of the midday meals program of AWCs. Telangana’s PDS currently procures rice via two mechanisms: custom milled rice (CMR) and “levy rice.” Via CMR, the Telangana government procures husked rice directly from farmers and pays mills to process it, whereas via “levy rice,” it is mandated that the government can purchase a portion of rice at a fixed minimum price from selected producers [48]. Using these two procurement approaches, low-income households in Telangana receive 6 kg of rice per household member from the PDS on a monthly basis [49].

While the PDS is a viable distribution channel to reach vulnerable population groups [39], our study shows that fortified rice could substantially increase intake of micronutrients women and children only if implemented under ideal conditions (i.e. that all rice consumed by women and children is fortified). If fortification were implemented, one could reasonably presume that some proportion of non-fortified rice (e.g. non-PDS rice or rice consumed outside the home) would be consumed by women and children, even if they received fortified rice as a take-home ration or as part of on-site supplementary feeding. Thus, our RDA estimates likely over-estimate the micronutrient intake that could be possibly realized by fortified rice. As our survey data does not enable us to estimate the proportion of non-fortified rice that could potentially be substituted with fortified rice, it is difficult to model alternative nutrient intake scenarios. PDS is also susceptible to changes in public policy [24]. In Telangana, there has recently been a change in the definition classifying households as “below poverty line,” which will enable a greater number of households to receive rice from the PDS [49]. Current and future changes to criteria permitting or restricting access to the PDS program should be considered if rice fortification is pursued.

To advance rice fortification in Telangana, a greater understanding of the micronutrient status of vulnerable groups in the general population, such as children and women of reproductive age, and the public distribution of rice is needed. Data on micronutrient deficiencies in Telangana are outdated, and more current data on the severity of micronutrient deficiencies in children and women are needed before a rice-fortification program can be justified. In particular, micronutrient deficiencies in individuals residing in low-income households should be explored, as these individuals would be the likely recipients of fortified rice distributed by the PDS. Due to considerable data gaps, the prevalence of the deficiency of all micronutrients added to fortified rice (e.g., iron, zinc, vitamin A, folate, and vitamin B12) should be thoroughly investigated prior to rice fortification (Table 3). A comprehensive assessment of the rice milling industry and distribution of PDS rice in Telangana is required. Such an assessment should detail the number of millers engaged by the PDS, the production capacity of these mills, and potential costs and coverage of rice fortification under varying scenarios of participation.

### Limitations

Because the survey used AWCs as the primary sampling unit and selected survey subjects only from AWC catchment areas, the results are not directly comparable to surveys where population-based sampling was done. Program data demonstrate that in most districts, 62%–78% of all children 6–35 months of age live in an AWC catchment area in their district. The exception is Hyderabad, a largely urban district, in which only one-third of children live in an AWC catchment area. Nonetheless, because the ICDS targets poor areas, the households eligible for inclusion in this survey may coincide somewhat with the target population for food fortification programs. Although the survey’s results are not representative of all households in Telangana, we found a similar coverage of iodized salt to previous studies in Andhra Pradesh and rice-consumption estimates compared to those produced by the Food and Agriculture Organization of the United Nations [50].

Another limitation of the study is the lack of more-accurate information, such as 24-hour dietary recall data, to calculate individual-level consumption of rice. Our method for estimating individual rice consumption was based on household-level rice purchases, and thus did not take into account rice consumed outside of the home. In addition, we assumed that rice consumption by household members was proportional to their caloric intake, as reflected by the Adult Male Equivalent. Thus, our calculations of rice consumed may under- or over-estimate the actual amount of rice consumed.

Lastly, the lack of data on the urinary iodine concentration of women and children is also a notable limitation. Though iodine delivered via a household's table salt is likely a key source of iodine, food consumed outside the home may also be source of iodine. This is particularly relevant for pregnant and lactating women and children 3–6 years of age residing in AWC catchment areas, as these groups are provided hot meals from the AWCs that contain a ration of rice, dahl, eggs, and vegetables [51], and likely contain iodized salt [14]. Particularly for pregnant and lactating women, the iodine received by those consuming the supplementary meals may provide a substantial amount of iodine to the diet.

## Conclusions

The coverage of adequately iodized salt in AWC catchment areas in Telangana is suboptimal, and increasing the adequacy of iodization of smaller salt manufacturers would help achieve universal salt iodization. Market-based rice fortification is not feasible in Telangana due to a large proportion of households producing their own rice and a highly fragmented rice distribution/milling industry. Fortified rice delivered through Telangana’s PDS system is a feasible approach to delivering fortified rice to the poorest households, but would only reach a small proportion of the households in AWC catchment areas and a small proportion of the total population in Telangana.

## Supporting Information

S1 File Questionnaire (English & Telegu). (PDF)
