# Correction: Coverage of Adequately Iodized Salt Is Suboptimal and Rice Fortification Using Public Distribution Channels Could Reach Low-Income Households: Findings from a Cross-Sectional Survey of Anganwadi Center Catchment Areas in Telangana, India

**DOI:** 10.1371/journal.pone.0163385

**Published:** 2016-09-15

**Authors:** James P. Wirth, Magali Leyvraz, Prahlad R. Sodani, Grant J. Aaron, Narottam D. Sharma, Bradley A. Woodruff

Reference 29 is cited in error in the published article. Reference 29 should be removed from the article. There is also an error in Reference 11 in the published article. The correct reference is: Leyvraz M, Wirth JP, Woodruff BA, Sankar R, Sodani PR, Sharma ND, et al. High Coverage and Utilization of Fortified Take-Home Rations among Children 6–35 Months of Age provided through the Integrated Child Development Services Program: Findings from a Cross-Sectional Survey in Telengana, India. PLoS ONE. Public Library of Science; 2016.

## References

[1] Wirth JP, Leyvraz M, Sodani PR, Aaron GJ, Sharma ND, Woodruff BA (2016) Coverage of Adequately Iodized Salt Is Suboptimal and Rice Fortification Using Public Distribution Channels Could Reach Low-Income Households: Findings from a Cross-Sectional Survey of Anganwadi Center Catchment Areas in Telangana, India. PLoS ONE 11(7): e0158554 doi:10.1371/journal.pone.0158554 27447925 10.1371/journal.pone.0158554 PMC4957802

