# Supplementary material for: Coverage of Adequately Iodized Salt Is Suboptimal and Rice Fortification Using Public Distribution Channels Could Reach Low-Income Households: Findings from a Cross-Sectional Survey of Anganwadi Center Catchment Areas in Telangana, India
Source: PLoS One. 2016 Jul 22;11(7):e0158554. doi: 10.1371/journal.pone.0158554 (PMC4957802; doi:10.1371/journal.pone.0158554)
Supplement: S1 File — (PDF) [file pone.0158554.s001.pdf]

IIHMR Health and Nutrition Survey

****

BACKGROUND INFORMATION

ప్రాథమిక సమాచారము

district: District name జిల్లా పేరు : ____________________

mandal: Mandal name మండలము పేరు : ____________________

psuname: PSU name పిఎస్‌యు పేరు : ____________________

psuid: PSU number పిఎస్‌యు నెంబరు |___|___|

psutype: Area type ప్రాంతము యొక్క రకము |__|Urban psutype=1 |__|Rural/tribal psutype=2

chname: Child name బిడ్డ పేరు : ____________________

chid: Child id బిడ్డ యొక్క గుర్తింపు నెం.. |___|___|

hhloc: Location of child's household బిడ్డ ఉంటున్న ఇంటి ప్రదేశము :____________________

****

TO BE FILLED IN BY INVESTIGATOR

ఇన్వెస్టిగేటర్ పూరించవలెను

iid: Investigator ID ఇన్వెస్టిగేటర్ గుర్తింపు నెం.: |___|___|

iname: Investigator name ఇన్వెస్టిగేటర్ పేరు :____________________

Investigator's signature ఇన్వెస్టిగేటర్ సంతకము :____________________

dateint: Date (DD/MM/YY) తేది (రోజు/నెల/సం॥) |___|___|/ |_1_|___|/|_1_|_4_|

****

FOR OFFICE USE ONLY

ఆఫీసు కొరకు మాత్రమే

sid: Supervisor: ____________________ సూపర్‌వైజర్ Signature: ____________________ సంతకము |___|___|

feid: Field editor: ____________________ ఫీల్డ్ ఎడిటర్ Signature: ____________________ సంతకము |___|___|

oeid: Office editor: ____________________ ఆఫీసు ఎడిటర్ Signature: ____________________ సంతకము |___|___|

d1id: Data entry op1: ____________________ డేటా ఎంట్రీ ఆపరేటర్1 Signature: ____________________ సంతకము |___|___|

d2id: Data entry op2: ____________________ డేటా ఎంట్రీ ఆపరేటర్2 Signature: ____________________ సంతకము |___|___|

****

obs: Please write here any other observations or comments (example: Refused or Absent)

మీ దృష్టికి వచ్చిన (గుర్తించిన) ఇతర అంశాలను ఇక్కడ వ్రాయండి (ఉదా : నిరాకరించుట లేదా ఎవరూ లేకపోవడం)

Consent form

Organisation: Indian Institute of Health Management Research (IHMR) Jaipur

Project: Coverage of the AP Foods' Bal Amrutham, distributed as a take-home ration through the ICDS system in Telangana

Introduction and details of the project: I am ______________________________, representing here from Indian Institute of Health Management Research, Jaipur for the study on coverage of the AP Foods' Bal Amrutham, distributed as a take-home ration through the ICDS system in Telangana funded by GAIN, Geneva. The main objective of the study is to determine the coverage of the AP Foods' product, called Bal Amrutham, distributed as a take-home ration to children below 3 years of age living in Telangana. I will be performing the following procedures on your child and his/her mother/care-giver.

Procedures: Measuring mid-arm circumference and oedema examination through observation and palpation of the sampled child. And measuring mid-arm circumference of the mother/care giver of the sampled child.

Sharing the Results and confidentiality: The results will be shared with you. It will also be presented/ disseminated at scientific or professional meetings/workshops or published in scientific journals. However, your child's identity will not be disclosed, the details and results will be maintained confidential.

Benefits: You will not have to pay anything for this, nor will you receive any monetary benefit for the same.

Participation: If you have read this form and have decided to participate in this project, please understand your participation is voluntary and you have the right to withdraw your consent or discontinue participation at any time without penalty. The interview will be administered in a setting that you deem comfortable.

Contact: In case of emergency, you can contact our field level staff at the number provided: Mr. Goutham Ghosh, Mobile No. 09829959664.

____

Signature of Investigator (on behalf of respondent)

సంస్థ : ఇండియన్ ఇన్‌స్టిట్యూట్ ఆఫ్ హెల్త్ మేనేజ్‌మెంట్ రిసెర్చ్ (ఐఐహెచ్ఎమ్ఆర్), జైపూర్

ప్రాజెక్టు : తెలంగాణ రాష్ట్రంలో ఐసిడిఎస్ ద్వారా ఎపి ఫుడ్స్ బాల అమృతం ఇంటికి తీసుకువెళ్ళే అనుబంధ ఆహారం (రేషన్) పథకం యుక్క అమలు

ప్రాజెక్టు గురించి పరిచయం మరియు వివరములు : నేను -----------------------------------, ఇండియన్ ఇన్‌స్టిట్యూట్ ఆఫ్ హెల్త్ మేనేజ్‌మెంట్ రీసెర్చి (ఐఐహెచ్ఎమ్ఆర్), జైపూర్ వారి తరుపున, తెలంగాణ రాష్ట్రంలో ఐసిడిఎస్ ద్వారా అమలులో ఉన్న ఎపి ఫుడ్స్ బాల అమృతం, ఇంటికి తీసుకువెళ్ళే అనుబంధ ఆహారం పథకం అమలుపై గెయిన్ (GAIN) జెనీవా వారి ఆర్థిక సహాయంతో అధ్యయనము నిర్వహిస్తున్నాము. ఈ స్టడీ యొక్క ముఖ్య ఉద్దేశ్యము ఏమనగా తెలంగాణ రాష్ట్రంలో 3 సంవత్సరాలలోపు పిల్లలకు ఇచ్చే అనుబంధ ఆహారం పథక అయినటువంటి ఎపి ఫుడ్స్ బాల అమృతం యొక్క కవరేజ్‌ను ఖచ్చితంగా తెలుసుకొనుట. నేను ఈ క్రింద తెలిపిన విధానాలను మీ బిడ్డ మరియు అతడు / ఆమె తల్లి లేదా సంరక్షకురాలిపై నిర్వహించబోతున్నాను.

విధానము : గుర్తించబడిన బిడ్డను పరిశీలన మరియు పరిస్పర్శన ద్వారా చేయి మధ్యస్థ చుట్టుకొలత కొలుచుట మరియు వాపు పరీక్ష చేస్తాము. మరియు గుర్తించబడిన బిడ్డ యొక్క తల్లి / సంరక్షకురాలి చేయి మధ్యస్థ చుట్టుకొలత తీసుకొనబడును.

ఫలితాల పంపిణి మరియు గోపత్య : ఫలితాలను మీతో పంచుకోవడం జరుగుతుంది. ఈ ఫలితాలను శాస్త్రీయ లేదా ప్రొపెషనల్ సమావేశాలు / వర్క్‌షాప్‌లలో ప్రదర్శించడం మరియు వ్యాప్తి చేయబడును లేదా శాస్త్రీయ పత్రికలలో ప్రచురించడం జరుగుతుంది. అయినప్పటికి, మీ బిడ్డ గురించి ఎవరికి తెలియ చేయ బడదు, వివరములు మరియు ఫలితములు గోప్యముగా ఉంచబడతాయి.

లాభములు / ఉపయోగములు : వీటి కొరకు మీరు మాకు ఏ విధమైన రుసుము చెల్లించక్కల్లేదు, లేదా మీకు ఏ విధమైన రుసుము చెల్లించబడదు.

పాల్గొనుట : మీరు ఈ ఫారమ్ చదివి సర్వేలో పాల్గొనాలనుకుంటే స్వచ్ఛంధంగా పాల్గొనవచ్చును, మరియు మీరు ఎప్పుడైనా ఈ సర్వే నుంచి ఎప్పుడైనా నిష్క్రమించవచ్చును లేదా నిలిపివేయమని కోరవచ్చు, దీనికి ఎటువంటి చర్య తీసుకొనబడదు. ఈ ఇంటర్వ్యూని మీకు వీలుగా / సౌకర్యంగా వుండే విధంగా నిర్వహించబడుతుంది.

సంప్రదింపుము : అత్యవసర సమయంలో ఈక్రింద తెలిపిన మా క్షేత్రస్థాయి సిబ్బందిని సంప్రదించవచ్చు

శ్రీ. గౌతమ్ ఘోష్, మొబైల్ నెం. 09829959664

____

ఇన్వెస్టిగేటర్ యొక్క సంతకము (సమాధానం ఇచ్చు వారి తరపున)

HOUSEHOLD ROSTER

Fill in the information about the sampled mother and the child:
ఎంపిక చేయబడిన తల్లి మరియు బిడ్డ యొక్క వివరములు ఈక్రింది నమూనాలో నింపవలెను

<table>
<tr><th rowspan="2"></th><th rowspan="2">Name
పేరు</th><th rowspan="2">Sex (M/F)
లింగము
(ఆడ/మగ)</th><th colspan="2">Age వయస్సు</th><th rowspan="2">Ever attended school or college?
ఎప్పుడైనా స్కూలు లేదా కాలేజికి ఎళ్ళారా</th><th rowspan="2">Currently attending school or college?
ప్రస్తుతము స్కూలు లేదా కాలేజికి వెళుతున్నారు</th><th rowspan="2">5 or more years of education?
ఐదు లేదా అంత కంటే ఎక్కువ విద్యా సం॥లు</th></tr>
<tr><th>Years
సం॥లు</th><th>Months
నెలలు</th></tr>
<tr><td>caregiver/ mother
తల్లి /
సంరక్షకులు(డు</td><td></td><td>|__|</td><td>|__|__|</td><td></td><td>|__|Yes |__|No</td><td>|__|Yes |__|No</td><td>|__|Yes |__|No</td></tr>
<tr><td>Child
బిడ్డ</td><td></td><td>|__|</td><td></td><td>|__|__|</td><td></td><td></td><td></td></tr>
</table>

Who are family members that live with you now or mostly live with you?
Complete one line of the roster for each remaining household member:
మీ కుటుంబ సభ్యులు ఎవరు సాధారణముగా లేదా ప్రస్తుతము మీతో కలసి నివసిస్తున్నారు ?
ప్రతి కుటుంబ సభ్యుని వివరాలు క్రమ సంఖ్యలో (ఒకరి తరువాత ఒకరి వివరాలు) నమోదు చేయండి :

<table>
<tr><th rowspan="2"></th><th rowspan="2">Name
పేరు</th><th rowspan="2">Sex (M/F)
లింగము
(ఆడ/మగ)</th><th colspan="2">Age (years OR months) వయస్సు (సం॥లు లేదా నెలలు)</th><th colspan="3">For persons aged > 5 years
5 సంవత్సరాల కంటే ఎక్కువ వయస్సు</th></tr>
<tr><th>Years
సం॥లు</th><th>Months (children <5) నెలలు
(5 నెలల లోపు పిల్లలు)</th><th>Ever attended school or college?
ఎప్పుడైనా స్కూలు లేదా కాలేజికి వెళ్ళారా</th><th>Currently attending school or college?
ప్రస్తుతము స్కూలు లేదా కాలేజికి వెళుతున్నారు</th><th>5 or more years of education?
ఐదు లేదా అంత కంటే ఎక్కువ విద్యా సం॥లు</th></tr>
<tr><td>1</td><td></td><td>|__|</td><td>|__|__|</td><td>|__|__|</td><td>|__|Yes |__|No</td><td>|__|Yes |__|No</td><td>|__|Yes |__|No</td></tr>
<tr><td>2</td><td></td><td>|__|</td><td>|__|__|</td><td>|__|__|</td><td>|__|Yes |__|No</td><td>|__|Yes |__|No</td><td>|__|Yes |__|No</td></tr>
<tr><td>3</td><td></td><td>|__|</td><td>|__|__|</td><td>|__|__|</td><td>|__|Yes |__|No</td><td>|__|Yes |__|No</td><td>|__|Yes |__|No</td></tr>
<tr><td>4</td><td></td><td>|__|</td><td>|__|__|</td><td>|__|__|</td><td>|__|Yes |__|No</td><td>|__|Yes |__|No</td><td>|__|Yes |__|No</td></tr>
<tr><td>5</td><td></td><td>|__|</td><td>|__|__|</td><td>|__|__|</td><td>|__|Yes |__|No</td><td>|__|Yes |__|No</td><td>|__|Yes |__|No</td></tr>
<tr><td>6</td><td></td><td>|__|</td><td>|__|__|</td><td>|__|__|</td><td>|__|Yes |__|No</td><td>|__|Yes |__|No</td><td>|__|Yes |__|No</td></tr>
<tr><td>7</td><td></td><td>|__|</td><td>|__|__|</td><td>|__|__|</td><td>|__|Yes |__|No</td><td>|__|Yes |__|No</td><td>|__|Yes |__|No</td></tr>
<tr><td>8</td><td></td><td>|__|</td><td>|__|__|</td><td>|__|__|</td><td>|__|Yes |__|No</td><td>|__|Yes |__|No</td><td>|__|Yes |__|No</td></tr>
<tr><td>9</td><td></td><td>|__|</td><td>|__|__|</td><td>|__|__|</td><td>|__|Yes |__|No</td><td>|__|Yes |__|No</td><td>|__|Yes |__|No</td></tr>
<tr><td>10</td><td></td><td>|__|</td><td>|__|__|</td><td>|__|__|</td><td>|__|Yes |__|No</td><td>|__|Yes |__|No</td><td>|__|Yes |__|No</td></tr>
</table>

11		___	___ ___	___ ___	___ Yes ___ No	___ Yes ___ No	___ Yes ___ No
12		___	___ ___	___ ___	___ Yes ___ No	___ Yes ___ No	___ Yes ___ No
13		___	___ ___	___ ___	___ Yes ___ No	___ Yes ___ No	___ Yes ___ No
14		___	___ ___	___ ___	___ Yes ___ No	___ Yes ___ No	___ Yes ___ No
15		___	___ ___	___ ___	___ Yes ___ No	___ Yes ___ No	___ Yes ___ No
16		___	___ ___	___ ___	___ Yes ___ No	___ Yes ___ No	___ Yes ___ No
17		___	___ ___	___ ___	___ Yes ___ No	___ Yes ___ No	___ Yes ___ No
18		___	___ ___	___ ___	___ Yes ___ No	___ Yes ___ No	___ Yes ___ No
19		___	___ ___	___ ___	___ Yes ___ No	___ Yes ___ No	___ Yes ___ No
20		___	___ ___	___ ___	___ Yes ___ No	___ Yes ___ No	___ Yes ___ No

Check the roster regarding completion!

re: What is your religion?
మీ యొక్క మతము ఏమిటి

Cross *one box only ...*
ఒక దానిని మాత్రమే ‘×’ తో గుర్తించండి

Hindu	హిందు	___ re=1
Muslim	ముస్లిమ్	___ re=2
Sikh	సిక్కు	___ re=3
Christian	క్రిస్టియన్	___ re=4
Jain	జైన్	___ re=5
Buddhist	బుద్ధిజమ్	___ re=6
None	ఏదీకాదు	___ re=7
Prefer not to say	చెప్పడానికి ఇష్టపడలేదు	___ re=8
Other ఇతరములు : ___________________________		___ re=9

MPI (ASSETS) DATA (1)

dhs1: Do you or any anyone in your household own this dwelling?
ఈ నివాస స్థలము మీ లేదా మీ కుటుంబ సభ్యులలో ఎవరిదైనా స్వంతమా ?

|__| Yes |__| No

dhs2: How many rooms in this household are used for sleeping?
ఈ గృహములో నిద్రించడానికి ఎన్ని గదులను ఉపయోగిస్తున్నారు ?

Number of rooms for sleeping నిద్రించడానికి వాడే గదుల సంఖ్య : |__|__|

mpi1: Does your household have electricity?
మీ గృహమునకు విద్యుత్ సరఫరా ఉందా ?

|__| Yes |__| No

mpi2: What fuel does your household mainly use for cooking?
ముఖ్యముగా మీ కుటుంబము ఏవిధమైన ఇంధనమును వంట చేసుకొనుటకు వినియోగిస్తున్నారు ?

Cross *one box only …*
ఒక దానిని మాత్రమే 'x' తో గుర్తించండి

Electricity	విద్యుత్	|__| mpi2=1
LPG / Natural gas	ఎల్‌పిజి / ప్రకృతి సిద్ధమైన ఇంధనము (నేచురల్ గ్యాస్)	|__| mpi2=2
Biogas (Gobar gas)	బయోగ్యాస్ (గోబర్ గ్యాస్)	|__| mpi2=3
Kerosene	కిరోసిన్ (గ్యాస్ నూనె)	|__| mpi2=4
Coal / lignite	బొగ్గు / లిగ్నైట్	|__| mpi2=5
Charcoal	కట్టెబొగ్గులు	|__| mpi2=6
Wood	కట్టెలు	|__| mpi2=7
Straw / shrubs / grass	పొదలు / గడ్డి	|__| mpi2=8
Agricultural crop waste	వ్యవసాయ ఆధారిత వ్యర్థాలు	|__| mpi2=9
Dung cakes	పిడకలు (ఆవు, గేదె పేడతో చేసినవి)	|__| mpi2=10
Other ఇతరములు : ______________________		|__| mpi2=99

MPI (ASSETS) DATA (2)

mpi3: Do you or anyone in your household have ... ?
మీరు లేదా మీ ఇంటిలో నివసిస్తున్న ఇతర / ఎవరైన కుటుంబ సభ్యులు ఈ క్రింద తెలిపినవి కలిగి ఉన్నారా ?

Prompt for each item. Record all items owned by household ...
ప్రతి గృహోపకరణమును గురించి తెలిపి, ఆ ఇంటిలో గల అన్ని గృహోపకరణములను గుర్తించండి .....

Put cross 'x' తో గుర్తించుము

A table?	టేబుల్	__ Yes	__ No	mpi3a
A chair?	కుర్చీ	__ Yes	__ No	mpi3b
A cot or bed?	మంచము	__ Yes	__ No	mpi3c
A mattress?	పరుపు	__ Yes	__ No	mpi3d
A pressure cooker?	ప్రెషర్ కుక్కర్	__ Yes	__ No	mpi3e
A water pump?	వాటర్ పంపు	__ Yes	__ No	mpi3f
An electric fan?	ఫ్యాన్	__ Yes	__ No	mpi3g
A sewing machine?	కుట్టుమిషన్	__ Yes	__ No	mpi3h
A watch or clock?	గోడ గడియారం లేదా చేతి గడియారం	__ Yes	__ No	mpi3i
A radio or transistor?	రేడియో లేదా ట్రాన్సిస్టర్	__ Yes	__ No	mpi3j
A television?	టివి	__ Yes	__ No	mpi3k
A mobile or telephone?	మొబైల్ ఫోన్ లేదా టెలిఫోన్	__ Yes	__ No	mpi3l
A bicycle, tricycle, rikshaw?	సైకిల్, మూడు చక్రాల సైకిల్, రిక్షా	__ Yes	__ No	mpi3m
A motorcycle, scooter, auto-rikshaw?	మోటార్‌బైక్, స్కూటర్, ఆటోరిక్షా	__ Yes	__ No	mpi3n
A car, truck, or jeep?	కారు, ట్రక్ లేదా జీప్	__ Yes	__ No	mpi3o
A tractor?	ట్రాక్టర్	__ Yes	__ No	mpi3p
A thresher?	త్రెషర్ / కోత యంత్రం	__ Yes	__ No	mpi3q
A refrigerator?	రిఫ్రిజిరేటర్	__ Yes	__ No	mpi3r
A computer, laptop or tablet ?	కంప్యూటర్, ల్యాప్‌టాప్ లేదా ట్యాబ్లెట్	__ Yes	__ No	mpi3s
A horse/cow/donkey/buffalo cart?	గుర్రం / ఆవు / గాడిద / ఎద్దులబండి ?	__ Yes	__ No	mpi3t
A bank or a post-office account?	బ్యాంకు లేదా పోస్టాఫీసులో ఖాతా	__ Yes	__ No	mpi3u

mpi4 : What is the main material of the floor of the dwelling?
మీ ఇంటి గచ్చును ముఖ్యముగా ఏవి వినియోగించి నిర్మించబడినది

Observation. ***Cross*** *one box only …*
గమనించండి. క్రింద తెలిపిన వాటిలో కేవలం ఒక దానిని 'x' తో గుర్తించండి

Mud / clay /earth	మట్టి	__	mpi4=1
Sand	ఇసుక	__	mpi4=2
Dung	పేడ	__	mpi4=3
Raw wood planks	ముడి చెక్క పలకలు	__	mpi4=4
Palm / bamboo	తాటి / వెదురు	__	mpi4=5
Brick	ఇటుక	__	mpi4=6
Stone	రాయి	__	mpi4=7
Parquet / polished wood	మెరుగు పెట్టబడిన చెక్కతో	__	mpi4=8
Vinyl / asphalt	తారు మరియు వినైల్	__	mpi4=9
Ceramic tiles	సిరామిక్ టైల్స్	__	mpi4=10
Cement	సిమెంట్	__	mpi4=11
Carpet (only)	కార్పెట్ మాత్రమే	__	mpi4=12
Polished stone / marble / granite	మెరుగుపెట్టబడిన రాయి / మార్బుల్ / గ్రానైట్	__	mpi4=13
Other ఇతరములు : ________________________		__	mpi4=99

dhs3: What is the main material of the roof of the dwelling?
మీ ఇంటి పైకప్పు ముఖ్యముగా దేనితో నిర్మించబడినది

Observation. Cross *one box only …*
గమనించండి. క్రింద తెలిపిన వాటిలో ఒక దానిని 'x' తో గుర్తించండి

No roof	పైకప్పు లేదు	|__|	dhs3=1
Thatch / palm leaf / reed / grass	తాటి / వెదురు / గడ్డి	|__|	dhs3=2
Mud	మట్టి	|__|	dhs3=3
Sod / mud and grass mixture	మట్టి మరియు గడ్డితో కలిపి	|__|	dhs3=4
Plastic / polythene sheeting	ప్లాస్టిక్ లేదా పాలిథిన్ షీట్	|__|	dhs3=5
Rustic mat	రస్టిక్ మ్యాట్	|__|	dhs3=6
Palm / bamboo	తాటి / వెదురు	|__|	dhs3=7
Raw wood planks / timber	చెక్క పలకలు / కలప	|__|	dhs3=8
Unburnt brick	కాల్చని ఇటుకలు	|__|	dhs3=9
Loosely packed stone	లూస్‌లి ప్యాక్డ్ స్టోన్	|__|	dhs3=10
Metal / GI	కంకర / జిఐ	|__|	dhs3=11
Wood	చెక్క	|__|	dhs3=12
Calamine / cement fiber	కేలమైన్ / సిమెంట్ ఫైబర్	|__|	dhs3=13
Asbestos sheets	ఆస్‌బెస్టాస్ రేకులు	|__|	dhs3=14
RCC / RBC / cement / concrete	ఆర్‌సిసి / ఆర్‌బిసి / సిమెంట్ / కాంక్రీట్	|__|	dhs3=15
Roofing shingles	పెంకులతో కట్టిన రూఫింగ్	|__|	dhs3=16
Tiles	టైల్స్	|__|	dhs3=17
Slate	పలకరాయి	|__|	dhs3=18
Burnt brick	కాల్చిన ఇటుక	|__|	dhs3=19
Other ఇతరములు : ________________________		|__|	dhs3=99

dhs4: What is the main material of the exterior walls of the dwelling?
మీ ఇంటి బయటి గోడలు ముఖ్యముగా ఏ విధమైన పదార్థములతో నిర్మించినారు ?

Observation. Cross *one box only …*
గమనించండి. క్రింద తెలిపిన వాటిలో ఒక దానిని 'x' తో గుర్తించండి

No walls	గోడలు లేవు	|__|	dhs4=1
Cane / palm / trunks / bamboo	కేన్ / తాటి / చెట్లు మోడు / వెదురు	|__|	dhs4=2
Mud	మట్టి	|__|	dhs4=3
Grass / reeds / thatch	గడ్డి / వెదురు / తాటి	|__|	dhs4=4
Bamboo with mud	వెదురు మట్టితో కలిపి	|__|	dhs4=5
Stone with mud	రాయి మట్టితో కలిపి	|__|	dhs4=6
Plywood	చెక్క (ప్లైఉడ్)	|__|	dhs4=7
Cardboard	అట్టలు	|__|	dhs4=8
Unburnt brick	కాల్చని (పచ్చి) ఇటుక	|__|	dhs4=9
Raw wood / reused wood	ముడి చెక్క / వినియోగించిన చెక్కను తిరిగి ఉపయోగించుట	|__|	dhs4=10
Cement / concrete	సిమెంట్ / కాంక్రీట్	|__|	dhs4=11
Stone with lime / cement	సున్నపురాయి మరియు రాళ్ళతో / సిమెంట్	|__|	dhs4=12
Burnt bricks	కాల్చిన ఇటుక	|__|	dhs4=13
Cement blocks	సిమెంట్ ఇటుక	|__|	dhs4=14
Wood planks / shingles	చెక్క ముక్కలు / పెంకులు	|__|	dhs4=15
GI / metal / asbestos sheets	జిఐ / కంకర / ఆస్‌బెస్టాస్ రేకులు	|__|	dhs4=16
Other ఇతరములు : ________________________		|__|	dhs4=99

WATER, SANITATION, AND HYGIENE (WASH) (1)

నీరు, శుభ్రత మరియు పరిశుభ్రత

w1: What is the main source of drinking water for you and your child?
మీకు మరియు మీ పిల్లలకు ముఖ్యమైన త్రాగు నీటి వనరు ఏది ?

***Cross one** box only …*
కేవలం ఒక దానిని ‘x’ తో గుర్తించండి

Piped water into dwelling	ఇంటిలోనికి పైపుల ద్వారా నీటి లభ్యత	|__|	w1=1
Piped water into yard / plot	పైపుల ద్వారా ఇంటి ఆవరణలోనికి నీటి లభ్యత	|__|	w1=2
Public tap / standpipe	పబ్లిక్ కుళాయి / మెయిన్ కనెక్షన్ నుండి తీసుకున్న నీరు	|__|	w1=3
Tube-well / Borehole	బోరు బావి	|__|	w1=4
Protected dug well	సురక్షిత బావి	|__|	w1=5
Unprotected dug well	ఆసురక్షిత బావి	|__|	w1=6
Protected spring	సురక్షిత స్ప్రింగ్	|__|	w1=7
Unprotected spring	అసురక్షిత స్ప్రింగ్	|__|	w1=8
Rainwater (collection system)	వర్షపు నీరు (నిల్వ చేయు విధానము ద్వారా)	|__|	w1=9
Tanker truck	ట్యాంకర్ ట్రక్	|__|	w1=10
Cart with small tank or drum	బండిపై చిన్న ట్యాంక్ లేదా డ్రమ్ము ద్వారా సరఫరా	|__|	w1=11
Surface water (river / dam / lake / pond / stream / canal / irrigation channel)	భూఉపరితలంపై లభించే నీరు (నది / ఆనకట్ట / సరస్సు / చెరువు / కాలువ / వ్యవసాయం కొరకు ఉపయోగించే నీటి కాలువ)	|__|	w1=12
Bottled water / poly-pack water	ప్లాస్టిక్ సీసాలలో నింపిన నీరు / ఫాలీప్యాక్ నీరు	|__|	w1=13
Other ఇతరములు :_____________________________		|__|	w1=99

w2a: Do you **usually** do anything to your drinking water to make it safer to drink?
త్రాగునీటిని సురక్షితము చేయడానికి సాధారణంగా మీరు ఏదైనా పద్ధతిని పాటిస్తారా ?

|__| Yes |__| No

If NO → GO TO w3
కాదు అయితే, ప్రశ్న w3 నుంచి అడగండి.

w2b: What do you **usually** do to the water to make it safer to drink?
నీటిని సురక్షితం చేయడానికి సాధారణంగా మీరు ఏమి చేస్తారు ?

Do not prompt. Probe 'Anything else?'. ***Cross all** boxes that apply...*
జవాబు అందించకండి. ఇంకా ఏమైనా అని అడుగుతూ, తెలిపినవన్ని ‘x’ తో గుర్తించండి

Boil	మరిగించుట	|__|	w2ba
Use alum	పటిక ఉపయోగిస్తాము	|__|	w2bb
Add bleach / chlorine tablets	బ్లీచింగ్ పౌడర్ / క్లోరిన్ మాత్రలు కలుపుట	|__|	w2bc
Strain it through a cloth	గుడ్డని ఉపయోగించి వడకట్టుట	|__|	w2bd
Use a water filter (ceramic / sand / composite / etc.)	వాటర్ ఫిల్టర్ ఉపయోగించుట (సిరామిక్ / మట్టి / కాంపోసిట్ మొదలగున్నవి)	|__|	w2be
Use electronic purifier	కరెంట్ ద్వారా నడిచే వాటర్ ప్యూరిఫైయర్‌ని ఉపయోగించుట	|__|	w2bf
Let it stand and settle	కదపకుండా మరియు నిలకడగా వుండే వరకు వదిలేస్తాము	|__|	w2bg
Don't know	తెలియదు	|__|	w2by
Other ఇతరములు :_____________________________		|__|	w2bz

WATER, SANITATION, AND HYGIENE (WASH) (2)

w3: What kind of toilet facility do members of your household usually use?
మీ ఇంటిలోని కుటుంబ సభ్యులు సాధారణంగా ఏ విధమైన మరుగుదొడ్డి సదుపాయాన్ని ఉపయోగిస్తున్నారు ?

*Do not prompt. **Cross** one box only …*
జవాబు అందించకండి, కేవలం ఒక దానిని 'x' తో గుర్తించండి

Flush or pour flush toilet to piped sewer system<br>మురుగునీరు పైపులైన్‌కి అనుసంధానించిన ఫ్లష్ లేదా పోర్‌ఫ్లష్ మరుగుదొడ్డి	|__|	w3=1
Flush or pour flush toilet to septic tank<br>సెప్టిక్ ట్యాంకుకు అనుసంధానించిన ఫ్లష్ లేదా పోర్‌ఫ్లష్ మరుగుదొడ్డి	|__|	w3=2
Flush or pour flush toilet to pit latrine<br>గొయ్యి మరుగుదొడ్డిలోనికి ఫ్లష్ లేదా పోర్‌ఫ్లష్	|__|	w3=3
Flush or pour flush toilet to somewhere else<br>మరెక్కడికైనా అనుసంధానింపబడిన ఫ్లష్ లేదా పోర్‌ఫ్లష్ మరుగుదొడ్డి	|__|	w3=4
Ventilated improved pit (VIP) / biogas latrine<br>గాలి ప్రవాహమునకు అనువైన గొయ్యి దొడ్డి / బయోగ్యాస్ దొడ్డి	|__|	w3=5
Pit latrine with slab<br>కాంక్రీట్‌తో వేయబడిన పైకప్పుగల గొయ్యి దొడ్డి	|__|	w3=6
Pit latrine without slab / open pit<br>పైకప్పు లేని గొయ్యి దొడ్డి	|__|	w3=7
Twin put / composting toilet ట్విన్ పుట్ / కంపోస్టింగ్ దొడ్డి	|__|	w3=8
Dry toilet తడి లేని మరుగుదొడ్డి	|__|	w3=9
No facilities / in the open / bush or field<br>ఏవిధమైన సదుపాయం లేదు ఆరుబయలు / పొదలు మరియు పొలములో	|__|	**w3=10**
Don't know తెలియదు	|__|	w3=88
Other ఇతరములు : __________________________	|__|	w3=99

If w3=10 (no facilities) → GO TO w5
ఒకవేళ **w3** = 10 అయితే (ఏవిధమైన సదుపాయం లేకపోతే) **w5** కి వెళ్ళండి

w4: Do you share this facility with other households?
మీరు ఇతర కుటుంబాలతో కలిసి ఈ సౌకర్యాన్ని పంచుకుంటారా ?

|__| Yes |__| No

w5: Please tell me all of the occasions when it is important for you to wash hands...
మీరు చేతులు కడుక్కోవడం ఏఏ సందర్భాలలో ముఖ్యమనుకుంటున్నారో తెల్పండి ?

*Do not prompt. Probe 'Anything else?'. Cross **all** that apply .*
జవాబు అందించకండి, ఇంకా ఏమైనా అని అడగండి, తెలిపినవన్ని 'x' తో గుర్తించండి.

Before eating తినడానికి ముందు	|__|	w5a
Before feeding a child పిల్లలకు తినిపించే ముందు	|__|	w5b
Before cooking / preparing food ఆహారము తయారు చేయడానికి / వండటానికి ముందు	|__|	w5c
After defecation / urination మల / మూత్ర విసర్జన తరువాత	|__|	w5d
After cleaning a child that has defecated / changing nappies / washing diaper<br>మల విసర్జన చేసిన బిడ్డను శుభ్రచేసిన తరువాత / నేపిలు మార్చిన తరువాత /డైపర్స్ ఉతికిన తరువాత	|__|	w5e
None mentioned / Don't know చెప్పలేదు / తెలియదు	|__|	w5y
Other ఇతరములు : ________________________	|__|	w5z

HOUSEHOLD FOOD SECURITY (1)

hfs1: In the last month did all members of your household get TWO FULL meals every day?
గడచిన నెలలో మీ కుటుంబములోని సభ్యులందరకి ప్రతి రోజు రెండు పూటలా కడుపునిండా భోజనం లభించిందా ?

|__| **Yes** |__| No

If YES → GO TO hfs3
అవును అయితే **hfs3**కి ఎళ్ళండి

hfs2: In the last month how many days did your household members **NOT** get TWO FULL meals every day?
గడచిన నెలలో ఎన్ని రోజులు మీ కుటుంబములోని సభ్యులందరకి రెండు పూటలా కడుపునిండా భోజనం లభించలేదు ?

Number of days రోజులు : |__|__|

hfs3: Do all members of your household get TWO FULL meals every day all year around?
మీ కుటుంబ సభ్యులందరికి సంవత్సరము పొడువునా రెండు పూటలా ఆహారం దొరుకుతుందా ?

Cross one *box only …*
కేవలం ఒక దానిని 'x'తో గుర్తించండి

Yes, every day of the year	అవును, సంవత్సరములోని ప్రతి రోజు	|__|	**hfs3=1**
Some days of the year	సంవత్సరములో కొన్ని రోజులు	|__|	hfs3=2
No, no days of the year	లేదు, సంవత్సరములోని ఏ రోజు కూడా దొరకడం లేదు	|__|	**hfs3=3**
Don't know / Don't remember	తెలియదు / గుర్తు లేదు	|__|	hfs3=4

If 'YES, EVERY DAY' (hfs3=1) or 'NO, NO DAYS' (hfs3=3) → GO TO d1a.
అవును అయితే *(hfs3=1)* లేదా కాదు *(hfs3=3)* అయితే నుండి *d1a* అడగండి

hfs4: What months of the year do the members of your household **NOT** get TWO FULL meals?
మీ కుటుంబ సభ్యులందరికి సంవత్సరములోని ఏఏ నెలలో రెండు పూటలా ఆహారం దొరకదు?

Cross the *months that apply.*
వర్తించిన నెలలన్నింటిని 'x'తో గుర్తించండి

January	జనవరి	|__|	hfs4a
February	ఫిబ్రవరి	|__|	hfs4b
March	మార్చి	|__|	hfs4c
April	ఏప్రిల్	|__|	hfs4d
May	మే	|__|	hfs4e
June	జూన్	|__|	hfs4f
July	జూలై	|__|	hfs4g
August	ఆగస్టు	|__|	hfs4h
September	సెప్టెంబరు	|__|	hfs4i
October	అక్టోబరు	|__|	hfs4j
November	నవంబర్	|__|	hfs4k
December	డిసెంబర్	|__|	hfs4l

SHORT BIRTH HISTORY (MPI MORTALITY)

******NOTE to Investigator: Sensitive Questions! Do not read the options! ******

***** పరిశోధకునిక గమనిక : సున్నితమైన ప్రశ్నలు ! జవాబులను (ఐచ్ఛికాలను) చదవకండి *****

The purpose of the short birth history (SBH) component is to determine whether any child that was recently born (in the three most recent births) to the respondent has died.

షార్టు బర్త్ హిస్టరీ (ఎస్‌బిహెచ్) యొక్క ఉద్దేశ్యము ఏమనగా, ఒకవేళ ఈ మధ్య కాలంలో (ఈ మధ్య కాలంలో పుట్టిన ముగ్గురు బిడ్డల) మీకు పుట్టిన పిల్లల లో ఎవరైనా చనిపోయారేమో తెలుసుకోవడము.

Tell the mother / caregiver : తల్లికి / సంరక్షకురాలుకి చెప్పండి :

I will be asking about your recent births.
మీకు ఈ మధ్య కాలంలో జరిగిన జననాల గురించి నేను అడగదలచుకున్నానని.

Ask the following questions : క్రింది ప్రశ్నలు అడగండి :

d1a: How long ago was your most recent birth?
ఈ మధ్య కాలంలో మీకు కాన్పు ఎప్పుడు జరిగింది ?

Please fill the time and unit boxes. టైమ్ మరియు యూనిట్ బాక్సును నింపండి.

Duration: If 'don't remember', write '88' — Duration:|__|__| d1aa
టైమ్ : గుర్తు లేకపోతే '88' వ్రాయండి

Unit: Day=1, Month=2, Year=3, Don't remember=8 — Unit: |__| d1ab
యూనిట్ : రోజు = 1, నెల = 2, సంవత్సరము = 3, గుర్తు లేదు = 8

If it is the child that is selected as the respondent child, select "Alive" in d1b and GOTO d2a.
బిడ్డ సమాధానం చెప్పే వారి బిడ్డ అయితే 'జీవించి వున్నారు' అని d1b లో వ్రాసి ప్రశ్న d2a అడగండి

d1b: Where is this child now? ****** DO NOT READ THE OPTIONS ******
ప్రస్తుతం ఈ బిడ్డ ఎక్కడ ఉన్నాడు / ఉన్నది ? *** జవాబులను (ఐచ్ఛికాలను) చదవకండి ***

Alive / Living with me or others	జీవించి / నాతో లేదా ఇతరులతో ఉన్నాడు	|__|	d1b=1
Not alive / Passed away / No more	జీవించి లేడు / చనిపోయాడు / ఇక లేడు	|__|	d1b=2
Don't know	తెలియదు	|__|	d1b=8

d2a: Did you have a birth before this most recent one?
ఈ కాన్పు కంటే ముందు బిడ్డకు జన్మనివ్వడం జరిగిందా ? |__| Yes |__| **No**

If NO → GO TO next module 'INFANT AND YOUNG CHILD FEEDING'
లేదు అయితే 'ఇన్‌ఫ్యాంట్ అండ్ యంగ్ చైల్డ్ ఫీడింగ్' మాడ్యూల్‌కి వెళ్ళండి

d2b: Where is this child now? ****** DO NOT READ THE OPTIONS ******
ఈ బిడ్డ ప్రస్తుతం ఎక్కడ ఉంటున్నాడు ? *** జవాబులను (ఐచ్ఛికాలను) చదవకండి ***

Alive / Living with me or others	జీవించి / నాతో లేదా ఇతరులతో ఉన్నాడు	|__|	d2b=1
Not alive / Passed away / No more	జీవించి లేడు / చనిపోయాడు / ఇక లేడు	|__|	d2b=2
Don't know	తెలియదు	|__|	d2b=8

d3a: Did you have a birth before that one?
దీని కంటే ముందు ఇంకో బిడ్డకు జన్మనిచ్చినారా ? |__| Yes |__| **No**

If NO → GO TO next module 'INFANT AND YOUNG CHILD FEEDING'
లేదు అయితే 'ఇన్‌ఫ్యాంట్ అండ్ యంగ్ చైల్డ్ ఫీడింగ్' మాడ్యూల్‌కి వెళ్ళండి

d3b: Where is this child now? ****** DO NOT READ THE OPTIONS ******
ఈ బిడ్డ ప్రస్తుతం ఎక్కడ ఉన్నారు ? *** జవాబులను (ఐచ్ఛికాలను) చదవకండి ***

Alive / Living with me or others	జీవించి / నాతో లేదా ఇతరులతో ఉన్నాడు	|__|	d3b=1
Not alive / Passed away / No more	జీవించి లేడు / చనిపోయాడు / ఇక లేడు	|__|	d3b=2
Don't know	తెలియదు	|__|	d3b=8

INFANT AND YOUNG CHILD FEEDING (IYCF)

iycf1: Is [NAME OF CHILD] currently breastfed?
బిడ్డ (పేరు)కు ప్రస్తుతం తల్లిపాలు పడుతున్నారా ? |__| Yes |__| No

iycf2a: Does [NAME OF CHILD] take any liquid other than breast milk?
బిడ్డ (పేరు) తల్లిపాలు కాకుండా ఇతర ఏమైనా ద్రవాహారం తీసుకుంటున్నాడా ?
|__| Yes |__| No

iycf2b: Does [NAME OF CHILD] take any solid or semi-solid food apart from breast milk?
బిడ (పేరు) తల్లిపాలతో పాటు ఘన లేదా మధ్యస్థ ఆహారం ఏమైనా తీసుకుంటున్నాడా ?
|__| Yes |__| No

iycf3: How many times was [NAME OF CHILD] fed mashed or pureed food or solid or semi-solid food as a meal or a snack since this time yesterday?
బిడ్డ (పేరు)కు గతరోజు / నిన్న ఈ సమయం నుండి మెత్తని, తురిమిన, ఘన, మధ్యస్థ ఆహారము భోజనముగా లేదా చిరుతిళ్ళ రూపంలో ః ఎన్నిసార్లు ఇవ్వడం / తినిపించడం జరిగింది ?
Number of times ఎన్నిసార్లు : |__|__| iycf3

MOTHER AND CHILD DIETARY DIVERSITY

Since this time yesterday, what food did you and [NAME OF CHILD] eat?
నిన్న ఇదే సమయం నుండి ఇప్పటి వరకు, మీరు మరియు మీ బిడ్డ (పేరు) ఏమి ఆహారం తీసుకున్నారు ?
Probe: "Did you have any of the following things to eat or drink" for items not mentioned
ఈక్రింది తెలిపిన కాకుండా ఇతర ఆహార పదార్థాలు ఏమైనా తిన్నారా లేదా త్రాగారా అని అడగండి.
**Check if mother/caregiver and child are vegetarian or not to avoid offense in dd10-12*
తల్లి / సంరక్షకురాలు మరియు బిడ్డ శాఖాహారులైతే వారు తప్పుగా భావిస్తారు గనుక *dd10-12*
Cross ALL boxes that apply!
వర్తించిన వాటన్నింటిని 'x' తో గుర్తించండి

		Caregiver	Child
**dd01**	Plain water? నీరు	|__|	|__|
**dd02**	Milk-based liquids (excluding breast milk) పాలతో తయారు చేసిన ద్రవాలు (చనుబాలు కాకుండా):<br>- Tinned, powdered or fresh milk, such as cow milk, goat milk, buffalo milk<br>- డబ్బా, పౌడర్ లేదా తాజా పాలు అనగా ఆవు, మేక, గేదెపాలు<br>- Infant formula such as Mother Dairy, Nan Pro (Nestlé), Lactogen<br>- చిన్న పిల్లల కోసం తయారు చేసిన మదర్ డైరీ, నాన్ ప్రో (నెస్లే), లాక్టోజెన్	|__|	|__|
**dd03**	Spices, condiments and other beverages : Curry, pickle, chili, tea or infusion, light soup, sweetened or flavoured water, sodas, malt drinks (Horlicks), coffee, janam ghutti, nimou, liquor, beer<br>మసాలా దినుసులు, సుగంధ ద్రవ్యాలు మరియు ఇతర పానీయాలు : కూర, పచ్చడి, కారం, టీ లేదా ఇన్ఫ్యూషన్ , తేలికైన సూప్, తీపి లేదా ఫ్లేవర్డ్ నీరు, సోడాలు, మాల్ట్ డ్రింక్స్ (హార్లిక్స్), కాఫీ, జనమ్‌గుట్టి, నిమ్మవు, లిక్కర్, బీర్	|__|	|__|
**dd04**	Any food made from grain such as millet (bajra), wheat, rice, maize, semolina, atta flour, maggi noodles, porridge, jau, chapati, Bal Amrutham<br>గింజల నుండి తయారు చేసిన ఏవిధమైన ఆహారము అనగా జొన్న, గోధుమ, వరి, మొక్కజొన్న, సెమోలినా, గోధుమ పిండి, మేగీ న్యూడిల్స్, పారిట్జ్, జావ, చపాతి, బాల అమృతం	|__|	|__|

**dd05**	Any food made from fruits or vegetables that have yellow or orange flesh such as carrots, kachar, red sweet potatoes, ripe mangoes, ripe papaya, balam kakdi, khadoo, kachri<br>పసుపు లేదా ఆరెంజ్ కలరు తొక్కలు కలిగిన కూరగాయలు లేదా పండ్ల నుంచి తయారు చేసిన ఏవిధమైన ఆహారము అనగా క్యారెట్, కచార్, చిలకడ దుంప, ముగ్గిన మామిడి పండ్లు, ముగ్గిన బొప్పాయి, బాలఎు కాకిడి, గుమ్మడి, పచ్చి మామిడికాయ	|__|	|__|
**dd06**	Any dark green leafy vegetables such as palak, methi, cholei, sarso, bathua, sejan, spring onion, radish leaves<br>ముదురు ఆకుపచ్చ రంగులో వుండే ఆకుకూరలు అనగా పాలక్, మెంతి, చొలాయి, ఆవ ఆకులు, బచ్చలి, సేజన్, స్ప్రింగ్ ఆనియన్, ముల్లంగి ఆకులు	|__|	|__|
**dd07**	Any food made from roots or tubers such as arbi, moli, jimmikand (white), white potatoes, white sweet potato, onions, ginger<br>రూట్స్ మరియు ట్యూబర్స్ ఉపయోగించి చేసిన ఆఎారము అనగా చేమ, ముల్లంగి, జిమ్మికండ్ (తెల్లవి), బంగాళా దుంపలు తెల్లవి, చిలకడ దుంప తెల్లవి, ఉల్లిపాయలు, అల్లం	|__|	|__|
**dd08**	Any food made from dhal, lentils, beans, Negal gram, channa, urad, peas, green gram, horse gram, besan, moong, soya bean, ker, copea, sattu, peanut paste, kichari, nuts, or seeds<br>పప్పులు ఉపయోగించి చేసిన ఆహారము అనగా పప్పులు, కాయ ధాన్యాలు, బీన్స్, నేగల్‌గ్రామ్, శెనగపప్పు, కంది పప్పు, పచ్చి బఠాణి, పెసరపప్పు, ఉలవలు, సెనగ, మినుము, సోయా బీన్, కేర్, కోపియ, సట్టు, వేంశెనగ పేస్టు, కిచారి, నట్స్, లేదా గింజలు	|__|	|__|
**dd09**	Any other fruits or vegetables such as apple, green mango, banana, pomegranate, lemon, guava, brinjal, tomatoes, beetroot, fali, tinda, kakri, parwal, gilki, peppers, singara, lal sag, lauki, toru, guar, gawanar, kakdi, cauliflower, cabbage, cucumbers, sweet corn, sangri, kudu, bjindi, coconut<br>ఇతర కూరగాయలు లేదా పండ్లు అనగా యాపిల్, మామిడి, అరటిపండు, దానిమ్మ, నిమ్మ, జామ, వంకాయ, టమోటా, బీట్‌రూట్, ఫాలి, టిండా, కాక్రీ, పార్వల్, గిల్కి, మిరియాలు, సింగాడ, లాల్ సేగ్, ఆనపకాయ, టోరు, గౌర్, గవనార్, క్యాలీఫ్లవర్, క్యాబేజి, కీర, జొన్న (తీపివి), సాంగ్రి, కుడు, బిజింది, కొబ్బరి	|__|	|__|
**dd10**	Liver, kidney, heart, or other organ meats of chicken, goat, lamb, sheep, beef<br>లివర్, కిడ్నీ, గుండె, లేదా కోడి, మేక, గొర్రె, పొట్టేళ్ళు, ఆవు యొక్క ఇతర అవయవాల ద్వారా లభించే మాంసము	|__|	|__|
**dd11**	Any meat such as chicken, goat, sheep, teetar, beef, pork<br>మాంసము అనగా కోడి, మేక, గొర్రె, టీటర్, ఆవు, పంది	|__|	|__|
**dd12**	Fresh or dried fish, shellfish, or seafood, crabs, frogs, lizards, snakes, grubs<br>తాజా లేదా ఎండ బెట్టిన చేప, షెల్‌ఫిష్ లేదా సముద్రఆహారం, పీతలు, కప్పలు, బల్లులు, పాములు, గ్రబ్స్	|__|	|__|
**dd13**	Milk-based products such as curd, lassi, buttermilk/chaach, paneer/cheese, mawa barfi, shrikhand, yoghurt, porridge with dairy<br>పాల ఉత్పత్తులతో తయారు చేసిన ఆహారము అనగా పెరుగు, లస్సి, బటర్‌మిల్క్ / మజ్జిగ, పనీర్ / చీజ్, మావా బర్ఫీ, శ్రీకంద్, తీపి పెరుగు (యోగార్ట్), పాలతో చేసిన పొరిడ్జ్	|__|	|__|
**dd14**	Eggs గ్రుడ్లు	|__|	|__|
**dd15**	Sugary foods such as biscuits, bon, toost, sweets, candies, sugar cane, cakes, chocolate<br>తీపి పదార్ధాలైనటు వంటి బిస్కట్స్, బన్స్, బూన్, టోస్టు, స్వీట్స్, క్యాండీస్, చెరుకు, కేకులు, చాక్లెట్లు	|__|	|__|
**dd16**	Any food made with oil, fat, coconut fat, butter or ghee<br>నూనె, ఫ్యాట్, కొబ్బరి ఫ్యాట్, వెన్న లేదా నెయ్యితో చేసిన ఏ విధమైన ఆహారము	|__|	|__|
**dd17**	Red palm oil ఎరుపు పామాయిల్	|__|	|__|

ICDS SERVICES (1)

icds1: Have you ever heard of the Anganwadi centers?
మీరు ఎప్పుడైనా అంగనవాడి సెంటర్ గురించి విన్నారా ?

|__| Yes |__| **No**

If NO → GO TO module 'BAL AMRUTHAM'
కాదు అయితే, 'బాల అమృతం' మాడ్యూల్ నుంచి అడగండి

icds2: Have you ever been to an Anganwadi center?
మీరు ఎప్పుడైనా అంగన్‌వాడి సెంటర్‌కు వెళ్ళారా ?

|__| **Yes** |__| No

If YES → GO TO icds4
అవును అయితే, icds4 ప్రశ్న నుండి అడగండి

icds3: Why have you never been to an Anganwadi center?
మీరు అంగన్‌వాడి సెంటర్‌కు ఎప్పుడూ ఎందుకు వెళ్ళలేదు ?

Do not prompt. Probe 'Anything else?'. ***Cross*** *all that apply.*
జవాబు అందించకండి, ఇంకా ఏమైనా అని అడుగుతూ, వర్తించినవన్నీ 'x'తో గుర్తించండి

Not willing or not interested వెళ్ళడానికి ఇష్టం లేదు	|__|	icds3a
No nearby facility / Too far / no transportation / cost of transportation దగ్గరలో అంగన్‌వాడి కేంద్రం లేదు / చాలా దూరం / రవాణా సౌకర్యం లేదు / రవాణాకి అయ్యే ఖర్చు	|__|	icds3b
Opening hours not convenient / Center not open తెరిచి ఉంచే సమయం అనుకూలంగా లేదు / అంగన్‌వాడి సెంటర్ తెరిచి ఉండడం లేదు	|__|	icds3c
Cost of services సేవలకు అయ్యే ఖర్చు	|__|	icds3d
Husband / family did not allow భర్త / కుటుంబ సభ్యులు అంగీకరించరు	|__|	icds3e
Class / Cast / Religion not welcome మతం / కులం / వర్గం అంగీకరించరు	|__|	icds3f
Don't know తెలియదు	|__|	icds3y
Other ఇతరములు : ______________________	|__|	icds3z

→ GO TO module 'BAL AMRUTHAM'
'బాల అమృతం' మాడ్యూల్ నుండి ప్రశ్నలు అడగండి

icds4: What services provided by the Anganwadi center have you ever used/received?
అంగన్‌వాడి కేంద్రంలో లభిస్తున్న సేవలలో మీరు వేటిని ఉపయోగించుకున్నారు/తీసుకున్నారు ?

Do not prompt. Probe 'Anything else?'. **Cross** all that apply.
జవాబు అందించకండి, ఇంకా ఏమైనా అని అడుగుతూ, వర్తించినవన్నీ 'x' తో గుర్తించండి

Supplementary food/take-home ration/hot cooked meals for child అదనపు ఆహారము / ఇంటికి తీసుకువెళ్ళే రేషన్ / వండి వడించే ఆహారము	|__|	icds4a
Daily hot cooked meal for pregnant woman/Lactating mother గర్భవతి/బాలింతలకు రోజూ వేడిగా వండి వడించే ఆహారము	|__|	icds4b
Immunization/vaccination వ్యాధి నిరోధక టీకాలు / వాక్సినేషన్	|__|	icds4c
Health check-up ఆరోగ్య పరీక్షలు	|__|	icds4d
Growth & weight monitoring of child బిడ్డ యొక్క పెరుగుదల మరియు బరువు పర్యవేక్షణ	|__|	icds4e
Early childhood care / pre-school education శిశు సంరక్షణ / ప్రాథమిక విద్య	|__|	icds4f
Education on health, family planning, nutrition, hygiene ఆరోగ్య విద్య, కుటుంబ నియంత్రణ, పోషణ, పరిశుభ్రత	|__|	icds4g
Avail family planning method (condoms, pills) కుటుంబ నియంత్రణ పద్ధతులు (కాండోమ్, పిల్స్) పొందటానికి	|__|	icds4h
Advice on antenatal/postnatal care, institutional delivery ప్రసవానికి ముందు / ప్రసవంతరువాత, ఆసుపత్రి ప్రసవాలపై తగిన సలహా	|__|	icds4i
Advice on disease prevention and control వ్యాధుల నియంత్రణ మరియు అరికట్టుటపై సలహా	|__|	icds4j
Don't know / Don't remember తెలియదు / గుర్తులేదు	|__|	icds4y
Other ఇతరములు : ______________________	|__|	icds4z

ICDS SERVICES (2)

icds5: How often do you usually go to the Anganwadi center?
ఎంత తరుచుగా మీరు అంగనవాడి సెంటర్‌కి వెళతారు ?

Cross *one box only …* కేవలం ఒక దానిని మాత్రమే 'x' తో గుర్తించండి

Not at all	అసలు వెళ్ళము	|__|	icds5=1
Almost daily	ప్రతిరోజు	|__|	icds5=2
At least once a week	కనీసం వారానికి ఒక రోజు	|__|	icds5=3
At least once a month	కనీసం నెలలో ఒక రోజు	|__|	icds5=4
Less often	ఎప్పుడైనా	|__|	icds5=5
Don't know / Don't remember	తెలియదు / గుర్తులేదు	|__|	icds5=6
Other ఇతరములు : ____________________		|__|	icds5=9

icds6: When was the last time you went to an Anganwadi center?
క్రితం సారి అంగన్‌వాడి సెంటర్‌కి ఎప్పుడు వెళ్ళారు ?

Please fill the time and unit boxes. సమయము మరియు యూనిట్ బాక్సులను నింపండి

Time: If 'don't remember', write '88'
సమయం : గుర్తులేకపోతే '88' వ్రాయండి — Duration: |__|__| icds6a

Unit: Day=1, Month=2, Year=3, Don't remember=8
యూనిట్ : రోజు=1, నెల = 2, సం॥ = 3, తెలియదు = 8 — Unit: |__| icds6b

icds7: What do you like about the Anganwadi centers?
అంగన్‌వాడి కేంద్రములో మీకు నచ్చినది ఏది ?

Do not prompt. Probe 'Anything else?'. ***Cross*** *all that apply.*
జవాబు అందించకండి, ఇంకా ఏమైనా అని అడుగుతూ, వర్తించినవన్నీ 'x'తో గుర్తించండి

Nearby facility	అందుబాటులో దగ్గరగా వున్నది	|__|	icds7a
Opening hours convenient	సౌకర్యవంతమైన కేంద్రము తెరచి ఉంచు వేళలు	|__|	icds7b
Health personnel /Anganwadi worker	ఆరోగ్య సిబ్బంది / అంగన్‌వాడి కార్యకర్త	|__|	icds7c
Waiting time short	వేచి వుండు సమయము తక్కువ	|__|	icds7d
Good quality of care /Availability of good services	నాణ్యమైన సంరక్షణ / మంచి ఆరోగ్యసేవలు లభించుట వలన	|__|	icds7e
Free services	ఉచిత సేవలు	|__|	icds7f
Presence of female provider at the facility	కేంద్రములో మహిళా కార్యకర్త యొక్క అందుబాటులో వుండుట వలన	|__|	icds7g
Nothing	ఏమీ లేదు	|__|	icds7h
Other ఇతరములు : ____________________		|__|	icds7z

icds8: What do you not like about the Anganwadi centers?
అంగన్‌వాడి కేంద్రములో మీకు నచ్చనది ఏమిటి ?

Do not prompt. Probe 'Anything else?'. ***Cross*** *all that apply.*
జవాబు అందించకండి, ఇంకా ఏమైనా అని అడుగుతూ, వర్తించినవన్నీ 'x'తో గుర్తించండి

No nearby facility / Too far from household	కేంద్రము దగ్గరలో లేదు / ఇంటి దగ్గర నుంచి చాలా దూరం	|__|	icds8a
Opening hours not convenient	కేంద్రము తెరచి ఉంచు వేళలు సౌకర్యవంతముగా లేవు	|__|	icds8b
Health personnel often absent	ఆరోగ్య సిబ్బంది తరచుగా సెలవులు పెడతారు	|__|	icds8c
Anganwadi worker is not so good	అంగన్‌వాడి కార్యకర్త మంచివారు కారు	|__|	icds8d
Waiting time too long	వేచి ఉండు సమయము చాలా ఎక్కువ	|__|	icds8e
Poor quality of care	నాణ్యమైన సంరక్షణ లభించదు	|__|	icds8f
Cost of services	సేవలు ఖర్చుతో కూడుకున్నవి	|__|	icds8g
No female provider at facility	కేంద్రము వద్ద మహిళా కార్యకర్త లేరు	|__|	icds8h
Class / Cast / Religion not welcome	మతం / కులం / వర్గం అంగీకరించరు	|__|	icds8i
Nothing	ఏమీ లేదు	|__|	icds7j
Other ఇతరములు : ____________________		|__|	icds7z

BAL AMRUTHAM (1)

cf1: Have you ever seen or heard of Bal Amrutham for children?
మీరు ఎప్పుడైనా పిల్లల కోసం బాల అమృతం గురించి విన్నారా ?

SHOW PICTURE |__| Yes |__| No
బొమ్మ చూపించండి

If NO → GO TO module '*RICE COVERAGE*'
లేదు అయితే, 'రైస్ కవరేజ్' మాడ్యూల్ నుంచి అడగండి

cf2: Where did you hear about it?
ఎక్కడ విన్నారు ?

Do not prompt. Probe 'Anything else?'. **Cross** all that apply.
జవాబు అందించకండి, ఇంకా ఏమైనా అని అడుగుతూ, వర్తించినవన్నీ '×' తో గుర్తించండి

Anganwadi center / worker అంగన్‌వాడి కేంద్రము / కార్యకర్త	|__|	cf2a
TV టివి	|__|	cf2b
Radio రేడియో	|__|	cf2c
Billboards / wallpapers / painted walls<br>బోర్డు, గోడ మీద అంటించే పోస్టరు ద్వారా / గోడ మీద బొమ్మలు	|__|	cf2d
Mobile van activation మొబైల్ వ్యాన్ ప్రచారం ద్వారా	|__|	cf2e
Village meetings గ్రామ సమావేశాలలో	|__|	cf2f
Private nursery / Pre-school worker<br>ప్రైవేట్ నర్సరీ / ప్రాథమిక పాఠశాల వర్కర్ ద్వారా	|__|	cf2g
Health clinic / health worker / IYCF mobiliser<br>హెల్త్ క్లినిక్ / ఆరోగ్య కార్యకర్త / ఐఎసిఎఫ్ మొబలైజర్	|__|	cf2h
Community leaders సమాజ నాయకులు	|__|	cf2i
Relative / friend / neighbor బంధువులు / స్నేహితులు / చుట్టుప్రకలవారు	|__|	cf2j
Store / pharmacy మందులు దుకాణం	|__|	cf2k
Market place సంత జరిగే ప్రదేశాలలో	|__|	cf2l
Don't know / don't remember తెలియదు / గుర్తు లేదు	|__|	cf2y
Other ఇతరములు : ______________________	|__|	cf2z

cf3: Have you ever received Bal Amrutham for before?
'బాల అమృతం ఇంతకు ముందు ఎప్పుడైనా తీసుకున్నారా ?

SHOW PICTURE |__| Yes |__| **No**
బొమ్మ చూపించండి

If NO → GO TO module '*RICE COVERAGE*'
ఒకవేళ కాదు అయితే, 'రైస్ కవరేజ్' నుంచి అడగండి

BAL AMRUTHAM (2)

cf4: When did you last receive Bal Amrutham for [NAME OF CHILD]?
(పేరు) కొరకు చివరగా బాల అమృతం ఎప్పుడు తీసుకున్నారు ?

Please fill the time and unit boxes. సమయము మరియు యూనిట్ బాక్సులను నింపండి

Duration: If 'don't remember', write '88' — Duration: |__|__| cf4a
సమయం : గుర్తులేకపోతే '88' వ్రాయండి

Unit: Day=1, Month=2, Year=3, Don't remember=8 — Unit: |__| cf4b
యూనిట్ : రోజు=1, నెల = 2, సం॥ = 3, తెలియదు = 8

If below or equal to 30 days (or one month), GO TO cf6.
30 రోజులు లేదా అంతకంటే తక్కువ అయితే, cf6 నుంచి అడగండి

cf5: Why did you not receive Bal Amrutham in the past month?
గత నెలలో 'బాల అమృతం ఎందుకు తీసుకు వెళ్ళలేదు ?

*Do not prompt. Probe 'Anything else?'. **Cross** all that apply.*
జవాబు అందించకండి, ఇంకా ఏమైనా అని అడుగుతూ, వర్తించినవన్నీ '×' తో గుర్తించండి

No money for transport రవాణా ఖర్చులకు డబ్బులు లేవు	|__|	cf5a
No time to go వెళ్ళడానికి టైమ్ లేదు	|__|	cf5b
Because of weather conditions వాతావరణ పరిస్థితుల కారణంగా	|__|	cf5c
Opening hours not convenient / Center not open<br>కేంద్రము తెరచి వుండు వేళలు అనుకూలంగా లేవు / కేంద్రము తెరచి లేదు	|__|	cf5d
Husband / family does not always allow<br>భర్త / కుటుంబ సభ్యులు అస్తమానం వెళ్ళడానికి అనుమతించరు	|__|	cf5e
Not necessary అవసరం లేదు	|__|	cf5f
Not customary సంప్రదాయము కాదు	|__|	cf5g
Other ఇతరములు : ______________________	|__|	cf5z

cf6: The last time you got Bal Amrutham, how much did you receive?
క్రితం సారి 'బాల అమృతం తీసుకున్నప్పుడు, ఎంత పరిమాణంలో పొందారు ?

If 'don't remember', write '88' — Number of 2.5kg packages: |__|__|
గుర్తులేకపోతే '88' వ్రాయండి — Other: ______________________________

cf7: The last time you got Bal Amrutham, how long did it last until you used it all?
క్రితం సారి తీసుకున్న 'బాల అమృతం ఎన్ని రోజుల వరకు ఉపయోగించారు ?

Please fill the time and unit boxes. సమయము మరియు యూనిట్ బాక్సు నింపండి

Time: If 'don't remember', write '88' — Duration: |__|__| cf7a
If 'other', write '99'
సమయం :***గుర్తులేకపోతే '88' వ్రాయండి , ఇతరము అయితే, '99' వ్రాయండి***

Unit: Day=1, Month=2, Year=3, 'don't remember'=8 — Unit: |__| cf7b
యూనిట్ : రోజు=1, నెల = 2, సం॥ = 3, తెలియదు = 8

Other **ఇతరము:** _______________

cf8: Who in the household currently eats Bal Amrutham and how often?
'బాల అమృతమును మీ ఇంటిలో ఎవరు మరియు ఎంత తరచుగా తింటున్నారు ?

Cross all. వర్తించినవన్నీ 'x' తో గుర్తించండి		Never ఎప్పుడూ లేదు	Sometimes కొన్నిసార్లు	Always ఎల్లప్పుడు
Index / interviewed child	ఇండెక్స్ / ఇంటర్వ్యూడ్ చైల్డ్	|___| cf8a=1	|___| **cf8a=2**	|___| **cf8a=3**
Me (mother / principal caregiver)	నేను (తల్లి / సంరక్షురాలు	|___| cf8b=1	|___| cf8b=2	|___| cf8b=3
Other young children (<5 y) in the household	ఇంటిలోని ఇతర చిన్న పిల్లలు (5 సం॥లోపు)	|___| cf8c=1	|___| cf8c=2	|___| cf8c=3
Other older children/adolescents (5-14y) in the household	ఇంటిలోని ఇతర పెద్ద / కౌమార (5-14 సం॥) పిల్లలు	|___| cf8d=1	|___| cf8d=2	|___| cf8d=3
Other adults in the household	ఇంటిలోని ఇతర పెద్దలు	|___| cf8e=1	|___| cf8e=2	|___| cf8e=3
Other ఇతరములు : ______________________		|___| cf8z=1	|___| cf8z=2	|___| cf8z=3
Don't know	తెలియదు			|___| cf8y=1

If the interviewed child(cf8a=1) never consume Bal Amrutham → GO TO cf9
ఒకవేళ ఇంటర్వ్యూడ్ చైల్డ్ *(cf8a=1)* ఎప్పుడూ బాల అమృతము వినియోగించకపోయినట్లయితే *cf9* వెళ్ళండి
If the interviewed child (cf8a=2 or 3) consumes Bal Amrutham → GO TO cf10
ఒకవేళ ఇంటర్వ్యూడ్ చైల్డ్ *(cf8a=2 or 3)* బాల అమృతము వినియోగించినట్లయితే *cf10* వెళ్ళండి

cf9: Why did [NAME OF CHILD] not consume it?
బిడ్డ (పేరు) ఎందుకు తినడం లేదు ?

Do not prompt. Probe 'Anything else?'. ***Cross*** *all that apply.*
జవాబు అందించకండి, ఇంకా ఏమైనా అని అడుగుతూ, వర్తించినవన్నీ 'x' తో గుర్తించండి

Food quality is not good	ఆహార నాణ్యత బాగాలేదు	|___| cf9a
Taste is not good	రుచికరంగా లేదు	|___| cf9b
Child does not like the product	బిడ్డకు ఈ ప్రాడెక్టు నచ్చలేదు	|___| cf9c
Gives loose motion to the child	బిడ్డకు విరేచనాలు అవటం వలన	|___| cf9e
Makes the child vomit	బిడ్డకు వాంతులు అవటం వలన	|___| cf9f
Rumor that it is not good for child	బిడ్డకు మంచిది కాదనే పుకారు వలన	|___| cf9g
Child is too young	బిడ్డ చిన్న పిల్ల అవటం వలన	|___| cf9h
Child eats the family meal	బిడ్డ కుటుంబము తినే ఆహారమే తీసుకుంటుంది	|___| cf9d
Other members of the household eat it	కుటుంబములోని ఇతర సభ్యులు తింటున్నారు	|___| cf9e
Don't know / Don't remember	తెలియదు / గుర్తులేదు	|___| cf9y
Other ఇతరములు : ____________________		|___| cf9z

→ **GO TO cf12** వెళ్ళండి

cf10: On an average day, how much Bal Amrutham **powder** does [NAME OF CHILD] eat?
బిడ్డ (పేరు) రమారమి / సుమారు రోజుకు ఎంత పరిమాణంలో 'బాల అమృతం పిండి' తింటుంది ?

Please fill the quantity and unit boxes.
పరిమాణము మరియు యూనిట్ 'బాక్సులలో' నింపండి

Quantity: |___|___|.|___|
Unit: |___|

Unit: Teaspoon (small) =1, tablespoon (big)=2, tea glass=3, cup=4, bowl=5, other=8
యూనిట్ : టీస్పూన్(చిన్నది) = 1, టేబుల్ స్పూన్(పెద్దది) = 2, టీ గ్లాసు = 3, కప్పు = 4, గిన్నె = 5, ఇతరములు = 8

If other ఇతరములు : ______________

cf11: What do you <u>usually and mainly</u> add to the Bal Amrutham powder before feeding it to the child?
మీ బిడ్డకు బాల అమృతం పిండి తినిపించే ముందు సాధారణంగా మరియు ముఖ్యముగా అందులో ఏమి కలిపి తినిపిస్తారు ?

Cross <u>one</u> *box only …*
కేవలం ఒక బాక్సును మాత్రమే 'x' తో గుర్తించండి

Family drinking water	ఇంటిలో త్రాగునీరు	|___|	cf11a
Hot water (boiled)	వేడి నీరు (మరిగించినవి)	|___|	cf11b
Warm water (not boiled)	గోరు వెచ్చని నీరు (మరిగించినవి కావు)	|___|	cf11c
Bottled water (packaged/mineral water) ప్లాసిక్ సీసాలలో లభించే నీరు (ప్యాక్ చేయబడిన / మినరల్ వాటర్)		|___|	cf11d
Hot milk (boiled)	వేడి పాలు (మరిగించినవి)	|___|	cf11e
Cold/warm/normal milk (not boiled)	చల్లని / గోరువెచ్చని / సాధారణ పాలు (మరిగించనివి)	|___|	cf11e
Nothing	ఏమీ లేదు	|___|	cf11f
Other ఇతరములు : ____________________________		|___|	cf11z

cf12: What do you like about Bal Amrutham?
బాల అమృతం లో మీకు నచ్చినది ఏమిటి ?

*Do <u>not</u> prompt. Probe 'Anything else?'. **Cross <u>all</u>** that apply.*
జవాబు అందించకండి, ఇంకా ఏమైనా అని అడుగుతూ, వర్తించినవన్నీ 'x' తో గుర్తించండి

Taste	రుచి	|___|	cf12a
Packaging	ప్యాకేజి	|___|	cf12b
It's free	ఉచితముగా లభిస్తుంది	|___|	cf12c
Good for the health of children	పిల్లల ఆరోగ్యానికి మంచిది	|___|	cf12d
Quality of the product is good	మంచి నాణ్యమైన ఉత్పత్తి	|___|	cf12e
Easy to prepare	తయారు చేయుట సులభము	|___|	cf12f
Name of the product	ఉత్పత్తికి ఉన్న మంచి పేరు	|___|	cf12g
Easily available	సులభంగా లభిస్తుంది	|___|	cf12h
Trustworthy source	నమ్మదగినది	|___|	cf12i
Nothing	ఏమీ లేదు	|___|	cf12j
Don't know	తెలియదు	|___|	cf12y
Other ఇతరములు : ____________________________		|___|	cf12z

cf13: What do you <u>not</u> like about Bal Amrutham?
బాల అమృతంలో మీకు నచ్చునది ఏమిటి ?
*Do <u>not</u> prompt. Probe 'Anything else?'. **Cross <u>all</u>** that apply.*
జవాబు అందించకండి, ఇంకా ఏమైనా అని అడుగుతూ, వర్తించినవన్నీ 'x' తో గుర్తించండ

Taste	రుచి	|___|	cf13a
Packaging	ప్యాకేజి	|___|	cf13b
Quality of the product is not good	ఉత్పత్తి నాణ్యమైనది కాదు	|___|	cf13c
Not easy to prepare	తయారు చేయడం కష్టము	|___|	cf13d
Name of the product	ఉత్పత్తికి అంత మంచిపేరు లేదు	|___|	cf13e
Not easily available	సులభంగా లభించదు	|___|	cf13f
Untrustworthy source	నమ్మకమైనది కాదు	|___|	cf13g
Nothing	ఏమీ లేదు	|___|	cf13h
Don't know	తెలియదు	|___|	cf13y
Other ఇతరములు : ____________________________		|___|	cf13z

BAL AMRUTHAM (5)

Cf14 : The last time you got Bal Amrutham, did you also receive EGGS?
క్రితం సారి 'బాల అమృతం తీసుకున్నప్పుడు, మీరు **గ్రుడు** కూడా తీసుకున్నారా ? |__| Yes |__| **No**

IF YES, How many? అవును అయితే, ఎన్ని **గ్రుడు** ?

If 'don't remember', write '88' Number of EGGS గ్రుడు : |__|__|
గుర్తులేకపోతే '88' వ్రాయండి Other: ______________________________

IF NO GOTO '*RICE COVERAGE*' **ఒకవేళ కాదు అయితే 'రైస్ కవరేజ్' నుంచి అడగండి**

Cf15: Who in the household currently eats these EGGS and how often?
మీ ఇంటిలో ఎవరు మరియు ఎంత తరచుగా ఈ **గ్రుడు** తింటున్నారు ?

Cross all. వర్తించినవన్నీ 'x' తో గుర్తించండి	Never ఎప్పుడూ లేదు	Sometimes కొన్నిసార్లు	Always ఎల్లప్పుడు
Index / interviewed child ఇండెక్స్ / ఇంటర్వ్యూడ్ చైల్డ్	|__| cf15a=1	|__| **cf15a=2**	|__| **cf15a=3**
Me (mother/principal caregiver) నేను (తల్లి / సంరక్షురాలు	|__| cf15b=1	|__| cf15b=2	|__| cf15b=3
Other young children (<5 y) in the household ఇంటిలోని ఇతర చిన్న పిల్లలు (5 సం॥లోపు)	|__| cf15c=1	|__| cf15c=2	|__| cf15c=3
Other older children/adolescents (5-14y) in the household ఇంటిలోని ఇతర పెద్ద / కౌమార (5-14 సం॥) పిల్లలు	|__| cf15d=1	|__| cf15d=2	|__| cf15d=3
Other adults in the household ఇంటిలోని ఇతర పెద్దలు	|__| cf15e=1	|__| cf15e=2	|__| cf15e=3
Other ఇతరములు : ________________________	|__| cf15z=1	|__| cf15z=2	|__| cf15z=3
Don't know తెలియదు			|__| cf15y=1

If the interviewed child(cf15a=1) never consume EGGS → GO TO cf16
ఒకవేళ ఇంటర్వ్యూడ్ చైల్డ్ *(cf15a=1)* ఎప్పుడూ గ్రుడు తినకపోయినట్లయితే *cf16* వెళ్ళండి
If the interviewed child (cf15a=2 or 3) consumes EGGS → GO TO rc1
ఒకవేళ ఇంటర్వ్యూడ్ చైల్డ్ *(cf15a=2 or 3)* గ్రుడు తింటున్నట్లయితే *rc1* వెళ్ళండి

Cf16: Why did [NAME OF CHILD] not consume it?
బిడ్డ (పేరు) ఎందుకు తినడం లేదు ?

*Do not prompt. Probe 'Anything else?'. **Cross all** that apply.*
జవాబు అందించకండి, ఇంకా ఏమైనా అని అడుగుతూ, వర్తించినవన్నీ 'x' తో గుర్తించండి

EGGS quality is not good	గ్రుడు నాణ్యత బాగాలేదు	|__| cf16a
Taste is not good	రుచికరంగా లేదు	|__| cf16b
Child does not like the EGGS	బిడ్డకు గ్రుడు నచ్చలేదు	|__| cf16c
Gives loose motion to the child	బిడ్డకు విరేచనాలు అవటం వలన	|__| cf16e
Makes the child vomit	బిడ్డకు వాంతులు అవటం వలన	|__| cf16f
Rumor that it is not good for child	బిడ్డకు మంచిది కాదనే పుకారు వలన	|__| cf16g
Child is too young	బిడ్డ చిన్న పిల్ల అవటం వలన	|__| cf16h
Child eats the family meal	బిడ్డ కుటుంబము తినే ఆహారమే తీసుకుంటుంది	|__| cf16d
Other members of the household eat it	కుటుంబములోని ఇతర సభ్యులు తింటున్నారు	|__| cf16e
Don't know / Don't remember	తెలియదు / గుర్తులేదు	|__| cf16y
Other ఇతరములు : ______________________		|__| cf16z

RICE COVERAGE (1)

rc1: What is the **main type** of rice do you usually use?
మీరు ఏ రకమైన బియ్యం సాధారణంగా వాడుతున్నారు ?

Cross *one box only.*
కేవలం ఒక బాక్సును 'x'తో గుర్తించండి

Par-boiled	ఉప్పుడు బియ్యం (పార్ బాయిల్డ్)	|__|	rc1=1
Raw	పచ్చి / దంపుడు / పాలిష్ చేయని బియ్యం	|__|	rc1=2
Quick-cook	తొందరగా ఉడికేది	|__|	rc1=3
Don't know	తెలియదు	|__|	rc1=8
Other ఇతరములు : ______________________________		|__|	rc1=9

rc2: Where does your household usually get rice from?
సాధారణంగా మీ కుటుంబం బియ్యం ఎక్కడ నుండి పొందుతారు ?

Cross *one box only.*
కేవలం ఒక బాక్సును 'x'తో గుర్తించండి

Home production	సొంత (ఇంటి) ఉత్పత్తి	|__|	**rc2=1**
Market / retailer / shop	మార్కెట్ / రిటైలర్ / షాప్	|__|	rc2=2
Local / village producer	స్థానికంగా / గ్రామంలో లభించేవి	|__|	rc2=3
PDS / Ration store	చౌక ధరల దుకాణము (పిడిఎస్) / రేషన్ స్టోరు	|__|	rc2=4
Gift / Relief	బహుమతి / సహాయం	|__|	rc2=5
Don't know	తెలియదు	|__|	rc2=8
Other ఇతరములు : ______________________________		|__|	rc2=9

If 'Home production' (rc2=1) → GO TO rc7
సొంత (ఇంటి) ఉత్పతి (rc2=1) అయితే (rc7) ప్రశ్న నుండి అడగండి

rc3: Do you usually get open or packaged rice?
మీరు సాధారణంగా విడిగా లభించే బియ్యం లేదా ప్యాక్ చేసిన బియ్యం తీసుకుంటారా ?

Cross *one box only.*
కేవలం ఒక బాక్సును మాత్రమే 'x'తో గుర్తించండి

Open	విడి బియ్యం	|__|	rc3=1
Packaged	ప్యాక్ చేసిన బియ్యం	|__|	rc3=2
Don't know	తెలియదు	|__|	rc3=8
Other: ఇతరములు ______________________________		|__|	rc3=9

rc4: What brand of rice do you usually get?
ఏ బ్రాండ్ బియ్యం మీరు సాధారణంగా ఉపయోగిస్తారు ?
Cross *one box only.*
కేవలం ఒక బాక్సును మాత్రమే 'x'తో గుర్తించండి

Annapurna	అన్నపూర్ణ	|__| rc4=1
Gold	గోల్డ్	|__| rc4=2
3-Star	3-స్టార్	|__| rc4=3
Heritage	హెరిటేజ్	|__| rc4=4
Superfine	సూపర్‌ఫైన్	|__| rc4=5
Don't know	తెలియదు	|__| rc4=8
Other ఇతరములు : ____________________________		|__| rc4=9

If 'don't know' (rc4=8) → GO TO rc6
ఒక వేళ తెలియదు (rc4=8) అయితే rc6 ప్రశ్నకు వెళ్ళండి

rc5: Why do you choose this brand?
మీరు ఈ బ్రాండ్ బియ్యం ఎందుకు ఎన్నుకున్నారు ?

*Do not prompt. Probe 'Anything else?'.**Cross** all that apply.*
జవాబు అందించకండి, ఇంకా ఏమైనా అని అడుగుతూ, వర్తించినవన్నీ 'x' తో గుర్తించండి

Good price / cheap / good value for money<br>మంచి ధర / చవక / డబ్బుకు తగ్గ విలువ	|__| rc5a
Good quality మంచి నాణ్యత	|__| rc5b
Easy availability సులభముగా లభిస్తుంది	|__| rc5c
Confidence / well known నమ్మకమైనది / బాగా తెలిసినది	|__| rc5d
Habit / Accustomed to brand అలవాటు / సర్దుకుపోయాము	|__| rc5e
Like the taste / texture రుచి నచ్చింది / రూపం	|__| rc5f
Easy preparation / convenience తయారు చేయుట సులభము / సౌలభ్యము	|__| rc5g
Like brand name బ్రాండ్ పేరు నచ్చింది	|__| rc5h
Like the way it is marketed మార్కెట్ చేసిన విధానము నచ్చింది	|__| rc5i
Don't know తెలియదు	|__| rc5y
Other ఇతరములు : ____________________________	|__| rc5z

rc6: This last time your household got rice, how much did you pay for it?
క్రితంసారి మీ కుటుంబం బియ్యం తీసుకున్నప్పుడు, ఎంత మొత్తం చెల్లించారు

If 'don't know', write '8888' Price: |__|__|__|__| Rupees
తెలియకపోతే '8888' గుర్తించండి

rc7: The last time your household got rice, how much did you get (for household consumption)?
క్రితం సారి మీ కుటుంబము బియ్యం తీసుకున్నప్పుడు, ఎంత పరిమాణంలో తీసుకున్నారు (కేవలం కుటుంబము యొక్క వినియోగం కోసం) ?

If 'don't know', write '8888.88' Quantity: |__|__|__|__|.|__|__| Kg
తెలియకపోతే '8888.88' గుర్తించండి

rc8: How long does this quantity usually last in your household?
ఈ మొత్తం పరిమాణం మీ కుటుంబానికి ఎంత కాలం వస్తుంది / సరిపోతుంది ?
Please fill the time and unit boxes. సమయము మరియు యూనిట్ బాక్సు నింపండి

Duration: If 'don't remember', write '88' Duration: |__|__| rc8a
సమయం : గుర్తులేకపోతే '88' వ్రాయండి
Unit: Day=1, Month=2, Year=3, Don't remember=8 Unit: |__| rc8b
యూనిట్ : రోజు=1, నెల = 2, సం॥ = 3, తెలియదు = 8

IODIZED SALT COVERAGE

is1: The last time your household purchased salt, how was it packaged?
మీరు క్రితంసారి కొన్న ఉప్పు ఎలా ప్యాక్ చేసి ఉంది ?

Cross one box only.
కేవలం ఒక బాక్సును మాత్రమే 'x'తో గుర్తించండి

Packaged salt	ప్యాకెట్‌లో ఉన్న ఉప్పు	|__|	is1=1
Open salt	విడి ఉప్పు	|__|	is1=2
Don't know	తెలియదు	|__|	**is1=8**

IF 'Don't know' (is1=8) or 'Open salt' (is1=2) → GO TO is4
ఒకవేళ సమాధానం తెలియదు అయితే ***(is1=8)***లేదా విడి ఉప్పు ***(is1=2)*** అయితే ***is4*** కి వెళ్ళండి

is2: What was the brand of the salt you got last time?
మీరు క్రితంసారి కొన్న ఉప్పు యొక్క బ్రాండ్ ఏమిటి ?

Cross one box only.
కవలం ఒక బాక్సును మాత్రమే 'x'తో గుర్తించండి

TATA	టాటా	|__|	is2=1
Aashirvaad	ఆశీర్వాద్	|__|	is2=2
Purty Plus	పూర్తి ప్లస్	|__|	is2=3
Ruchi Gold	రుచి గోల్డ్	|__|	is2=4
Saffola	సఫోలా	|__|	is2=5
Nirma	నిర్మా	|__|	is2=6
Amma Hastam	అమ్మ హస్తం	|__|	is2=7
Mahesh	మహేష్	|__|	is2=8
Don't know	తెలియదు	|__|	**is2=88**
Other ఇతరములు : ______________________________		|__|	is2=99

IF 'Don't know' (is2=88) → GO TO is4
ఒకవేళ సమాధానం తెలియదు అయితే ***(is2=88) is4*** కి వెళ్ళండి

is3: Does the [NAME OF SALT BRAND] have an iodization logo or has 'iodized' or 'iodine' written on the package?
మీరు కొన్న ఉప్పు ప్యాకెట్‌పై (బ్రాండ్ పేరు) అయోడైజేషన్ లోగో లేదా 'అయోడైజ్డ్' లేదా 'అయోడిన్' అని వ్రాసి ఉన్నదా ?

Check package. |__| Yes |__| No |__| Package not available
ప్యాకెట్ ఉంటే పరీక్షించండి ప్యాకెట్ అందుబాటులో లేదు

is4: May I take a small sample of your salt? |__| Yes |__| No
మీరు అనుమతిస్తే నమూనా కోసం నేను కొంచెం ఉప్పు తీసుకోవచ్చా ?

Take one tablespoon of salt.
ఒక టీస్పూన్ ఉప్పు తీసుకోండి
If YES → STICK label here
అవును అయితే, లేబుల్‌ని ఇక్కడ అంటించండి
If NO → Write in reason
కాదు అయితే, కారణం వ్రాయండి

STICK LABEL HERE
లేబుల్‌ని ఇక్కడ అంటించండి

MOTHER AND CHILD HEALTH AND NUTRITION DATA

<table>
<tr><th colspan="2">Caregiver / Mother</th></tr>
<tr><td>fefol1

fefol2</td><td>NOTE: SHOW iron tablet

Did you(the mother)receive iron tablets during your pregnancy with this child?
ఈ బిడ్డ గర్భములో ఉన్నప్పుడు మీరు (తల్లి) ఐరన్ మాత్రలు తీసుకున్నారా ? |__|Yes |__|No |__|Don't know

If 'No' or 'Don't know' → GO to muacm1
ఒకవేళ 'కాదు' లేదా 'తెలియదు' అయితే muacm1 కి వెళ్ళండి
Did you/the mother take all the iron tablets you received during your pregnancy with this child?
ఈ బిడ్డ గర్భములో ఉన్నప్పుడు మీరు / తల్లి తీసుకున్న అన్ని ఐరన్ మాత్రలు వేసుకున్నారా ?

|__|Yes |__|No |__|Don't know</td></tr>
<tr><td>muacm1

muacm2</td><td>NOTE: Take MUAC on the left arm గమనిక : ఎమ్‌యుఎసి ఎడమ చేతికి తీసుకోండి
Enter '555' and don't measure MUAC, if respondent is a male
ఒకవేళ సమాధానం ఇచ్చు వారు మగవారు అయితే '555' వ్రాయండి మరియు MUAC ని కొలవ వలసిన అవసరం లేదు
Enter '666' if MUAC tape is too small (arm too big for tape)
ఎమ్‌యుఎసి చాలా పెద్దగా వుంటే '666' వ్రాయండి
Enter '777' if mother refused |__|__|__| mm
తల్లి అంగీకరించకపోతే '777' వ్రాయండి

Cross one box only.|__|Bare skin |__| Light clothes |__|Thick clothes
కేవలం ఒక దానిని 'x'తో గుర్తించండి

If mother MUAC < 230 mm → REFER!
తల్లి ఎమ్‌యుఎసి <230 ఎమ్ఎమ్ అయితే రెఫర్ చేయండి</td></tr>
<tr><th colspan="2">Child</th></tr>
<tr><td>hc</td><td>Does the child have a mother child health card?
బిడ్డకు మాతాశిశు ఆరోగ్య కార్డు ఉన్నదా ?
|__|Yes |__| No |__|Don't know
తెలియదు</td></tr>
<tr><td>diar</td><td>In the past 14 days (2 weeks), has [NAME OF CHILD] had diarrhea?
(పేరు) గడచిన 14 రోజులలో (2 వారాలు) నీళ్ళ విరేచనాలతో బాధపడ్డారా ?
|__|Yes |__|No |__|Don't know</td></tr>
<tr><td>vomi</td><td>In the past 14 days (2 weeks), has [NAME OF CHILD] vomited?
(పేరు) గడచిన 14 రోజులలో (2 వారాలు) వాంతులు చేసుకున్నారా ?
|__|Yes |__|No |__|Don't know</td></tr>
<tr><td>vas</td><td>NOTE: SHOW vitamin A syrup విటమిన్ ఏ ద్రవము చూపించండి
Did [NAME OF CHILD] take vitamin A syrup in the past 6 months?
బిడ్డ (పేరు) గడచిన 6 నెలలలో విటమిన్ ఎ ద్రవము తీసుకున్నాడా ?
|__|Yes |__|No |__|Don't know</td></tr>
<tr><td>oedema</td><td>Oedema on both feet present?
వాపు రెండు పాదములలో ఉన్నదా ?
If oedema YES → Refer! |__|Yes |__|No |__|Refused
ఒకవేళ వాపు ఉన్నచో రెఫర్ చేయండ</td></tr>
<tr><td>bcg</td><td>BCG scar (check on left arm)
బిసిజి మచ్చ ఎడమ చేతిపై టీకా గుర్తు చూడండి
|__|Yes |__|No |__|Refused</td></tr>
</table>

**muacc1**<br><br><br><br><br><br><br>**muacc2**	***NOTE:*** *Take MUAC on the left arm* గమనిక : ఎమ్‌యుఎసి ఎడమ చేతికి తీసుకోండి __ __ __<br>*Enter '777' if refused* **ఒకవేళ అంగీకరించకపోతే '777' వ్రాయండి** |__|__|__| mm<br><br>***Refer child to health facility if:*** ఈ క్రింది పరిస్థితులలో బిడ్డను ఆరోగ్య కేంద్రానికి రెఫర్ చేయండి<br>***<6 months and MUAC < 110 mm*** **ఆరు నెలలోపు మరియు ఎమ్‌యుఎసి <110 ఎమ్ఎమ్**<br>***>6 months and MUAC < 115 mm*** **ఆరు నెలలపైన మరియు ఎమ్‌యుఎసి <115 ఎమ్ఎమ్**<br><br>*Cross one box only.* |__|Bare skin |__| Light clothes |__|Thick clothes<br>ఒక దానిని 'x'తో గుర్తించండి.

******THANK YOU*****
